# Differential Gene Expression in the EphA4 Knockout Spinal Cord and Analysis of the Inflammatory Response Following Spinal Cord Injury

**DOI:** 10.1371/journal.pone.0037635

**Published:** 2012-05-22

**Authors:** Kathryn M. Munro, Victoria M. Perreau, Ann M. Turnley

**Affiliations:** Department of Anatomy and Neuroscience, Centre for Neuroscience Research, The University of Melbourne, Parkville, Australia; Center of Ophtalmology, Germany

## Abstract

Mice lacking the axon guidance molecule EphA4 have been shown to exhibit extensive axonal regeneration and functional recovery following spinal cord injury. To assess mechanisms by which EphA4 may modify the response to neural injury a microarray was performed on spinal cord tissue from mice with spinal cord injury and sham injured controls. RNA was purified from spinal cords of adult EphA4 knockout and wild-type mice four days following lumbar spinal cord hemisection or laminectomy only and was hybridised to Affymetrix All-Exon Array 1.0 GeneChips™. While subsequent analyses indicated that several pathways were altered in EphA4 knockout mice, of particular interest was the attenuated expression of a number of inflammatory genes, including Arginase 1, expression of which was lower in injured EphA4 knockout compared to wild-type mice. Immunohistological analyses of different cellular components of the immune response were then performed in injured EphA4 knockout and wildtype spinal cords. While numbers of infiltrating CD3+ T cells were low in the hemisection model, a robust CD11b+ macrophage/microglial response was observed post-injury. There was no difference in the overall number or spread of macrophages/activated microglia in injured EphA4 knockout compared to wild-type spinal cords at 2, 4 or 14 days post-injury, however a lower proportion of Arginase-1 immunoreactive macrophages/activated microglia was observed in EphA4 knockout spinal cords at 4 days post-injury. Subtle alterations in the neuroinflammatory response in injured EphA4 knockout spinal cords may contribute to the regeneration and recovery observed in these mice following injury.

## Introduction

The Eph receptors are the largest family of tyrosine kinases [1], [2] and together with their ligands, the ephrins, they play a major role in modulation of numerous biological functions. They are expressed in a wide variety of tissues during development and in the adult, including in the central nervous system (CNS). Eph-ephrin signalling affects numerous processes in the developing CNS, largely related to cell adhesion/repulsion and cytoskeletal changes, including acting as axon guidance cues in the developing CNS, steering growth cones to their correct locations. EphA4 expression is vital for the correct establishment of spinal cord circuitry as demonstrated by the phenotype of EphA4 knockout mice [3] and is expressed within the developing spinal cord, where it has a critical role in the guidance of corticospinal tract axons [4].

Within the adult rodent spinal cord, EphA4 protein has been localised to axons of the dorsal funiculus [5], substantia gelatinosa [5], and glial cells within the white matter [5]. Weak expression has been observed in some motor neurons [6] and grey matter astrocytes [7]. EphA4 expression is markedly upregulated in the adult spinal cord following injury. EphA4 knockout mice display significant axonal regrowth by six weeks post-spinal cord injury (SCI), while histological differences between injured EphA4 knockout and wild-type mice are already evident as early as 4 days post-injury [6]. Further, in wild-type mice, blocking of the EphA4 receptor promotes axonal regeneration and functional recovery following SCI [8].

Ephs and ephrins are also associated with immune function and inflammation [9]. Expression of Ephs and ephrins can be upregulated by pro-inflammatory mediators in cell lines *in vitro*, and the changing expression of Ephs and ephrins may play a role in leukocyte movement into sites of inflammation by affecting the adhesion of leukocytes, and altering vascular permeability by changing the adhesion of endothelial cells [10] and CNS vascular formation [11]. Numerous Ephs and ephrins are expressed in endothelial cells, as well as leukocytes including monocytes and lymphocytes [10] and Eph receptor activation can regulate T lymphocyte adhesion [12]. EphA4 expression has been detected in subpopulations of CD4+ [13], [14] and CD8+ T lymphocytes [14], B lymphocytes [15], [16] and platelets [17], [18]. In the CNS, inflammatory cells in lesions from multiple sclerosis patients were found to express EphA4, as well as a number of other Ephs and ephrins [19].

A significant non-CNS phenotype observed in EphA4 knockout mice relates to their immune system. The thymic epithelium does not develop normally in these mice; by 4 weeks of age, the thymus of knockout mice is significantly smaller (with respect to body size) than seen in their wild-type littermates, leading to altered T cell development [20]. Therefore, while EphA4 knockout mice are in general healthy and their most obvious phenotype is a hopping gait, numerous physiological processes are subtly altered in these mice – not only in the CNS, but also in the vascular and immune systems.

EphA4 upregulation has been localised to reactive astrocytes following SCI in mice [6] and rats [7] and following brain injury in the marmoset [21] and rat [22], as well as regulating astrocyte reactivity *in vitro* [22], [23]. Low levels of EphA4 protein have been localised to axons near the lesion site in injured mice [6] and a similar observation has been made in injured rats: while EphA4 mRNA levels did not change in neurons of the motor cortex following injury, over time EphA4 protein accumulated in the injured axon stumps adjacent to the spinal cord lesion [7]. Following SCI in rats, EphA4 has additionally been detected in motor neurons [24].

There are a number of altered mechanisms which potentially contribute to the axonal regeneration and functional recovery seen in EphA4 knockout mice. The rationale behind performing the original hemisection experiments described above [6] was that the removal of a repulsive guidance cue from the lesion environment would result in less inhibition of axons attempting regrowth. However, many factors contribute to secondary damage and lack of regeneration following CNS injury and the extensive regeneration in EphA4 knockout mice suggested other factors may be contributing to this beneficial outcome, in addition to the removal of a source of axonal repulsion. These additional factors may include decreased astrocytic gliosis, altered blood flow or other vascular alterations, and a differential inflammatory response.

As a constitutive knockout mouse, the absence of EphA4 during development may lead to compensatory alterations in the expression of other genes, which may also impact injury response. Furthermore, EphA4 is expressed in multiple tissues, both within and outside the CNS. While EphA4 is an important axon guidance molecule and is upregulated in astrocytes following injury, less is known about its expression in other CNS and peripheral cell types relevant to SCI such as endothelial and immune cells. Gene expression profiling, using microarrays or other high-throughput technologies, is a powerful discovery tool for identifying differences in biological processes between tissues and has been widely used in neurosciences, particularly in models of CNS injury. In this study we used genome wide expression profiling to compare differential gene expression in wild-type and EphA4 knockout mice following SCI, to identify mechanisms regulated by EphA4 through which functional recovery may be regulated.

## Methods

### Mice

All animal work was approved by the University of Melbourne Anatomy & Cell Biology, Neuroscience, Pathology, Pharmacology, and Physiology Animal Ethics Committee in accordance with the Australian Code of Practice for the Care and Use of Animals for Scientific Purposes, approval number 0703762. Male and female EphA4 knockout mice and their wild-type littermates were bred on a C57/Bl6 background and were aged between 10 and 14 weeks. The generation of EphA4 knockout mice is described in Dottori et al [3]. Mice were group housed in standard cages with free access to food and water and were kept on a 12 hour light/dark cycle.

### Spinal Cord Injury Surgery and Mouse Care

Mice were anaesthetised with an intraperitoneal (IP) injection of ketamine (100 µg/g body weight; Ketamil, Troy Laboratories, Aust.) and xylazine (13.5 µg/g body weight; Xylazil, Troy Laboratories) in sterile phosphate buffered saline (PBS). Experimental mice were given a lumbar spinal cord hemisection: two to three vertebral laminae at T13-L1 were removed and the left side of the spinal cord was sectioned manually with a fine microsurgical knife (Coherent Scientific, Aust.). The spinal cord was cut twice in the same place to ensure complete section. Control mice underwent a laminectomy only. After surgery the wound was closed with small metal clips (Becton Dickinson, USA) applied using a skin applicator (World Precision Instruments, USA).

Analgesia was provided by IP injection of Carprofen (5 µg/g body weight; Rimadyl, Pfizer, UK) immediately following anaesthesia and 24 hours post-surgery, subcutaneous application of Lignocaine (Troy Laboratories) at the time of surgery, and the addition of paracetamol to drinking water (2 mg/ml water; liquid Panadol, GlaxoSmithKline, Aust.) for the remainder of the experiment. 0.9% saline (NaCl in sterile H_2_O) equivalent to 2% of body weight was administered IP before surgery, two hours post-surgery, and later as recommended by animal house staff. Mice were culled if they displayed weakness in their right leg, rapid weight loss, or any signs of lethargy or distress. All efforts were made to minimise animal numbers and animal discomfort.

### RNA Extraction and Microarray Hybridisation

Twelve mice were used for the microarray experiment: EphA4 and wild-type mice were given a spinal cord hemisection (*n* = 3 per genotype), or laminectomy only (*n* = 3 per genotype). Each genotype group was gender-balanced with two males and one female in each group. At 4 days post-injury mice were culled via CO_2_ asphyxiation. After laminectomy, 6 millimetres of tissue centred on the injury epicenter or equivalent area in control mice was manually dissected from the left (injured) half of the spinal cord. Tissue was immediately snap frozen on a mixture of isopentane (Chem-Supply, Aust.) and dry ice and stored at −80°C. RNA was extracted by homogenising each sample in 1 ml of TRIzol Reagent (Invitrogen, Aust.) according to the manufacturer’s protocol. Chloroform (Chem-Supply) was used in the phase separation, and 2-propanol (Sigma-Aldrich) in RNA precipitation. RNA was washed in 75% ethanol (from Molecular Biology Grade absolute ethanol, Sigma-Aldrich) and the RNA pellet was air dried briefly before being resuspended in RNase-free H_2_O. RNA was further purified using the RNeasy Micro Kit (Qiagen, Aust.) according to the manufacturer’s protocol and stored at −80°C. Samples were processed by the Australian Genome Research Facility (www.AGRF.org.au) where quality was assessed on an Agilent Bioanalyser 2100 before 1 µg RNA was hybridised to Affymetrix Mouse All-Exon ST Array GeneChips™, with RNA from a single mouse spinal cord hybridised to an individual GeneChip™.

### Bioinformatic Analysis

The data discussed in this publication have been deposited in NCBI’s Gene Expression Omnibus [25] and are accessible through GEO Series accession number GSE34430 (http://www.ncbi.nlm.nih.gov/geo/query/acc.cgi?acc=GSE34430).

Expression data was loaded into Partek Genomics Suite 6.5 (Partek, Missouri, USA) to assess the quality of the data and perform statistical analyses. Affymetrix CEL files were imported using the Robust Multi-Chip Average (RMA) algorithm. Affymetrix library files used in conjunction with Partek Genomics Suite were the V1 release version for the Mouse Exon 1.0 ST Array, and annotation files were from NetAffx (www.affymetrix.com). Core probesets only were selected and a gene-level summary analysis was conducted. Only relevant CEL files were loaded into Partek for analyses using only sham-operated or only injured samples; all files were loaded to conduct analyses of all samples, and all samples were normalised before conducting hierarchical clustering analysis. Few genes passed the stringent threshold for False Discovery Rate; therefore a p-value cut-off of 0.01 and a fold-change cut-off of 1.2 were used for all analyses to minimise the risk of generating false positive results.

Several programs were used to further analyse the lists of differentially expressed genes generated with the significance level and fold-change settings above, and place them in a functional context. Differentially expressed gene lists were entered into GoMiner [26] (discover.nci.nih.gov/gominer), a bioinformatics enrichment tool which uses data in the Gene Ontology database (www.geneontology.org) to identify gene ontology groups which are significantly over-represented in gene lists. Gene ontology categories were allocated a p-value, and we selected a p-value cut-off of 0.05. (The GoMiner settings used were:organism = mouse; data sources = all; evidence code = 3.) Significant gene ontology groups containing only one differentially expressed gene were disregarded to reduce the likelihood of false positives. Approximately 10% of entered genes were not recognised by GoMiner.

### Histology

Post-injury timepoints included were 2, 4, 14 and 42 days. To minimise animal number and suffering, naïve controls were used to compare to the multiple post-injury timepoints. Mice were culled with a lethal injection of pentobarbitone sodium (Lethabarb, Virbac, Aust., 150 mg) and transcardially perfused with 0.1 M phosphate-buffered saline (PBS) and 4% paraformaldehyde (PFA, Sigma-Aldrich, Aust.) in PBS. Spinal cord tissue was post-fixed in 4% PFA for two hours, rinsed in PBS, and placed in increasingly concentrated sucrose solutions (10%, 20% and 30%) in PBS. Spinal cords were left in 30% sucrose overnight, then immersed in Tissue-Tek O.C.T. compound (Sakura Finetek, USA) and snap-frozen in isopentane (Ajax Finechem, Aust.) cooled in liquid nitrogen. Spinal cords were sectioned at 10 µm on a Leica cryostat onto Superfrost Plus slides (Lomb Scientific, Aust.) in either the transverse or horizontal plane, and stored at −80°C until used. Every tenth slide from each spinal cord was stained with haematoxylin and eosin to identify the lesion site and assess tissue morphology. The rostro-caudal mid-point of lesioned tissue was deemed the injury epicenter for subsequent analyses.

### Immunohistochemistry

Cryosections were thawed at room temperature for at least 1 hour, rinsed in PBS, and incubated for a minimum of 30 minutes in blocking solution. For the majority of experiments, the blocking solution used was PBS containing 2% normal goat serum (NGS, Invitrogen, Aust.), 2% foetal calf serum (FCS, Thermo Scientific, Aust.) and 0.2% Tween-20 (Ajax Finechem). For Arginase 1 labelling, the blocking solution used was PBS containing 2% bovine serum albumin (BSA, Sigma-Aldrich) and 0.2% Tween-20. Sections were incubated with primary antibody diluted in blocking solution overnight at room temperature; these included rat anti-CD11b (Millipore, 1∶500), goat anti-Arginase 1 (Santa Cruz, 1∶250), and rabbit anti-CD3 (Abcam, 1∶500). Sections were rinsed in PBS three times for a minimum of 5 minutes, then incubated with a fluorophore-tagged secondary antibody diluted in PBS for 1 hour at room temperature. Secondary antibodies included goat anti-rat Alexa Fluor 488 (Invitrogen, 1∶500), donkey anti-goat Cy3 (Jackson ImmunoResearch, 1∶500) and donkey anti-rabbit Alexa Fluor 594 (Invitrogen, 1∶500). For Arginase 1 and CD11b double-labelling, sections were incubated with the secondary antibody for Arginase 1 before being incubated with the secondary antibody for CD11b, to avoid cross-reactivity. Cell nuclei were counterstained with DAPI (Sigma-Aldrich). Secondary antibody specificity was determined by omitting the primary antibody.

### Histological Analysis

#### Analysis of CD11b+ area in horizontal sections

CD11b immunostaining for macrophages and activated microglia was quantitated in a 1 mm rostro-caudal area encompassing the injury (500 µm rostral and caudal to the injury epicentre). Between 3 and 6 horizontal cryosections spaced 100 µm apart were analysed from uninjured (naïve) mice (n = 3 wild-type, n = 2 knockout) and hemisectioned mice at 2 days (n = 3 per genotype), 4 days (n = 3 per genotype) and 14 days (n = 2 per genotype) post-injury. The horizontal sections used were close to the midline and displayed both grey and white matter in intact areas. Images were taken on an Olympus IX81 microscope at x10 magnification with minimal alteration of imaging parameters between sections. The images were coded for blinded analysis, and the area of CD11b staining as a proportion of total tissue area on the injured side of the spinal cord was determined using the thresholding function in ImageJ (Rasband, W.S., ImageJ, U.S. National Institutes of Health, Bethesda, Maryland, USA, http://imagej.nih.gov/ij, 1997–2011). Additionally, the area of CD11b immunoreactivity was calculated in the following rostral and caudal fields from the epicentre within each section: 0 to 100 µm, 100 to 200 µm, 200 to 300 µm, 300 to 400 µm and 400 to 500 µm.

#### Analysis of CD11b+ area in transverse sections

CD11b immunoreactivity was quantitated in a 200 µm area centred around the lesion; 3 transverse cryosections spaced 100 µm apart were used with the middle section through the injury epicentre. Tissue was analysed from hemisectioned mice at 2 days (n = 3 per genotype), 4 days (n = 6 per genotype) and 14 days (n = 3 per genotype) post-injury. Tiled images taken on a confocal microscope at x40 magnification were compiled, coded for blinded analysis, and the area of positive staining as a proportion of total tissue area on the left hand side of the spinal cord was determined using the thresholding function in Image.

#### CD11b density counts

The density of CD11b+ cells was analysed in transverse sections within 200 µm of the injury epicentre (as above). Images were taken on a confocal microscope at x40 magnification and coded for blinded analysis. Cells were counted in areas throughout the injured region and the density of cells converted to cells/100 µm^2^. 3 fields were counted in tissue from hemisectioned mice at 2 days (n = 3 per genotype) and 14 days (n = 3 per genotype) post-injury and 8 fields from mice at 4 days post-injury (n = 6 per genotype). CD11b+ cells were counted when they co-localised with the DAPI nuclear counterstain.

#### Arginase 1-positive macrophage/activated microglia counts

The proportion of Arginase 1 immunoreactive ces which co-localised with CD11b+ macrophages/activated microglia was counted in transverse sections within lesioned tissue. Tissue was analysed from hemisectioned mice at 2 days (n = 3 per genotype), 4 days (n = 6 per genotype) and 14 days (n = 3 per genotype) post-injury. Tiled images taken of the entire injured left hand side of the spinal cord were taken on a confocal microscope at x40 magnification, and coded for blinded analysis. A minimum of 12 fields were counted from 2 days post-injury samples due to low density of activated microglia/macrophages, and a minimum of 8 fields were counted from 14 days post-injury samples. In 4 days post-injury samples, a minimum of 4 fields were selected from each of the ‘inner’ areas, closer to the midline of the spinal cord where macrophages were densely packed, as well as the ‘edge’ areas, where macrophages were dispersed among areas of necrotic tissue. CD11b+ cells with a DAPI nuclear counterstain were counted as either Arginase 1-positive or Arginase 1-negative.

### Statistics

For histological analyses, statistics were generated using Student’s t-test in GraphPad Prism (version 4.03, GraphPad Software, Inc.) and values are represented as mean ± standard error of the mean (SEM). Results were considered statistically significant at p<0.05.

## Results

### Quality Control of RNA and Microarray Samples

Assessment of RNA integrity on the Agilent Bioanalyser 2100 indicated an average RNA Integrity Number (RIN) of 9.5, thus RNA samples provided for the microarray were of high quality (Fig. S1A). The expression data was entered into Partek Genomics Suite where quality control analyses were performed. Principal Components Analysis (PCA) mapping indicated that the greatest variation between spinal cord samples was due to treatment, i.e. between sham-operated and injured samples (Fig. S1B). There was no obvious clustering based on genotype, gender, or RNA extraction date. No samples were identified as biological or technical outliers, therefore all samples were included in the subsequent bioinformatic analysis.

### Differential gene expression in sham-operated EphA4 knockout and wild-type spinal cords

To identify underlying gene expression differences between EphA4 knockout and wild-type spinal cords, a one-way Analysis of Variance (ANOVA) of sham-operated samples only was conducted (with genotype as factor). 119 genes were differently expressed between control EphA4 knockout and wild-type mice; 56 genes had a decreased expression in EphA4 knockout compared to wild-type spinal cords, while 63 had an increased expression.

Enrichment analysis using GoMiner was conducted to identify altered biological processes, molecular functions or cellular components involving more than one gene, to provide a more robust and comprehensive exploration of the data. Twenty four gene ontology categories were significantly over-represented in the gene list when applying a *p*-value cut-off of 0.05 (Table 1). Gene ontology categories were contained within 3 ontology domains: Biological Process, Molecular Function and Cell Component. The significant gene ontology categories containing the highest number of differentially expressed genes were ‘intracellular organelle’ and ‘organelle’ (56 genes), ‘intracellular membrane-bounded organelle’ and ‘membrane-bounded organelle’ (50 genes), ‘nucleus’ (35 genes), ‘cellular macromolecule biosynthetic process’ and ‘macromolecule biosynthetic process’ (23 genes), ‘transcription’ (19 genes) and ‘regulation of gene expression’ (19 genes).

**Table 1:** Comparison of sham-operated wild-type and EphA4 knockout spinal cords: gene ontology groups over-represented in list of differentially expressed genes.

Biological Process	p-value	Molecular Function	p-value
Regulation of pH	0.0058	Enzyme activator activity	0.0021
Monovalent inorganic cation		GTPase activator activity	0.0201
Homeostasis	0.0089	GTPase regulator activity	0.0392
Mitochondrion organization	0.0145	Nucleoside-triphosphatase regulator	
Regulation of transcription	0.0317	Activity	0.0419
Transcription	0.0346		
Regulation of cell morphogenesis		**Cellular Component**	**p-value**
Involved in differentiation	0.0361	Nucleus	0.0083
Regulation of gene expression	0.0375	Intracellular organelle	0.0143
Cellular macromolecule biosynthetic		Organelle	0.0145
Process	0.0408	Cell junction	0.0175
Macromolecule biosynthetic process	0.0420	Intracellular membrane-bounded	
Positive regulation of apoptosis	0.0442	Organelle	0.0275
Positive regulation of programmed		Membrane-bounded organelle	0.028
cell death	0.0456	Synapse	0.0362
Positive regulation of cell death	0.0463	Proteasome complex	0.0423

(GoMiner analysis: significance level of p<0.05).

### Differential gene Expression in Injured EphA4 Knockout and Wild-type Spinal Cords

To identify genes differentially expressed between genotypes following CNS injury, a one-way ANOVA was performed comparing injured groups only: 71 genes differentially expressed between genotypes passed a cut-off of *p*<0.01 and a fold change of ≥1.2 (Table S1). Arginase 1 narrowly missed this *p*-value threshold (p = 0.0164), however it was identified as a candidate gene for further analysis due to having the largest fold change (fold change 3.11) in the dataset.

**Figure 1:**
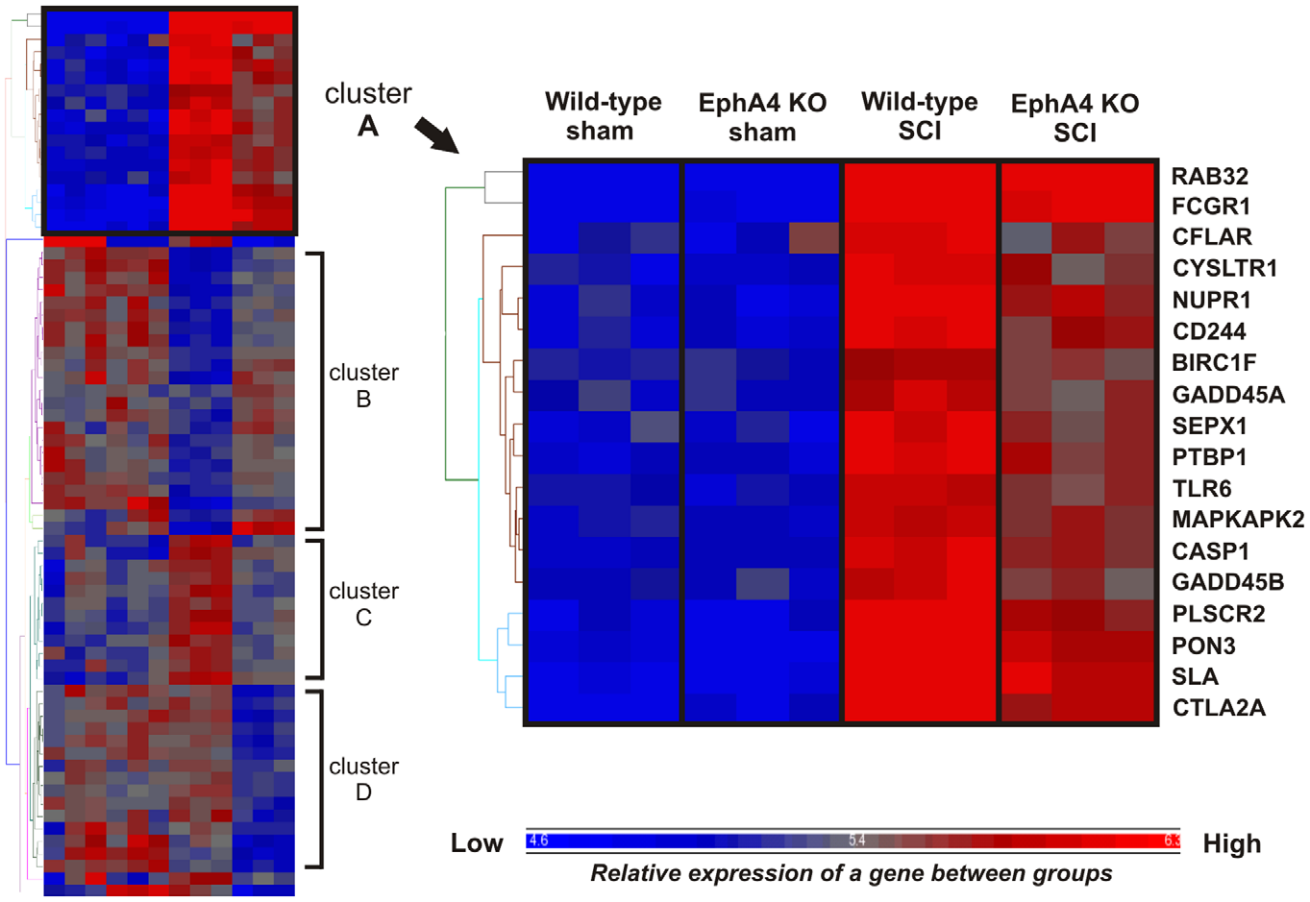
Hierarchical clustering of differentially expressed genes generated by comparison of injured wild-type and EphA4 knockout samples. Hierarchical clustering identified subsets of differentially expressed genes with similar expression profiles across groups. A cluster was chosen for further analysis (enlarged, cluster A); expression of genes in this cluster was relatively low in both sham-operated wild-type and EphA4 knockout samples and increased after injury in both genotypes. However the upregulation in injured EphA4 knockout samples was attenuated compared to injured wild-type samples. A number of genes with this expression profile were related to inflammation or immune function. KO = knockout.

Expression profiles of the 71 differentially expressed genes were then analysed across all four treatment groups using hierarchical clustering on expression data from all samples (Fig. S2). This identified groups of genes with similar expression profiles across all treatment groups. There were four distinct clusters containing genes with similar expression profiles, denoted clusters A to D (Fig. 1). Cluster A includes genes with a relatively low level of expression in sham-operated samples of both genotypes and upregulated expression in both genotypes following SCI. However the post-injury increase in gene expression was attenuated in EphA4 knockout compared to wild-type samples. A number of these genes are related to inflammation or immune function. These include Fc receptor, IgG, high affinity I (*Fcgr1*), CASP8 and FADD-like apoptosis regulator (*Cflar*), Cysteinyl leukotriene receptor 1 (*Cysltr1*), Nuclear protein 1 (*Nupr1*), CD244 natural killer cell receptor 2B4 (*Cd244*), Toll-like receptor 6 (*Tlr6*), MAP kinase-activated protein kinase 2 (*Mapkapk2*), Caspase 1 (*Casp1*), Src-like adaptor (*Sla*) and Cytotoxic T lymphocyte-associated protein 2 alpha (*Ctla2a*). The expression profile of genes in cluster A is shared by the gene Arginase 1 which is also related to inflammation, being expressed in a subset of macrophages. Arginase 1 was previously identified as a gene of biological interest based on its relatively large expression difference between injured wild-type and EphA4 knockout samples. The combination of its large fold change, its association with inflammation, and the similarity between its expression profile and that of other differentially expressed genes related to inflammation and immune function, led to the selection of Arginase 1 for further *in vivo* analysis. This analysis was conducted alongside a comparison of the general extent of inflammatory infiltration in injured wild-type and EphA4 knockout spinal cords.

Validation of changes in gene expression was performed by use of immunohistochemical localisation rather than RT-PCR. RT-PCR validation requires the use of control genes. Ideally at least three different control genes should be used, and they should be expressed at about the same level of the target gene to be validated and also not be differentially expressed in the biological system being examined. For this project we investigated the use of a number of standard control genes (Gapdh, Hprt1, Actb, B2 microglobulin, and Tbp). However all these genes were either previously known to be differentially expressed in CNS tissue following injury or were differentially expressed in our microarray data and so no appropriate reference point could be reliably established.

### Histology of Wild-type and EphA4 Knockout Spinal Cords Following Injury

To visualise the injury site and determine whether there was an attenuation of the post-injury inflammatory response in EphA4 knockout mice compared to wild-type mice, the presence of inflammatory cells (T cells and macrophages/activated microglia) were primarily examined *in vivo* at 4 days post-injury, to parallel the design of the microarray analysis and additionally at 2 and 14 days post-injury. The general morphology of hemisectioned spinal cords was examined using haematoxylin and eosin stained cryosections and no gross histological differences between genotypes were observed (Fig. S3).

### T Cell Infiltration is Low in Both Wild-type and EphA4 Knockout Spinal Cords Following Hemisection

**Figure 2:**
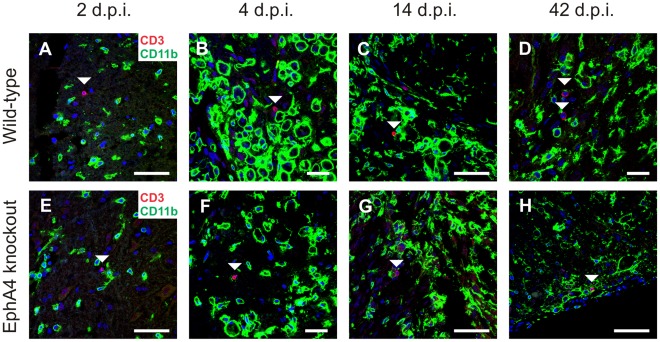
T cell infiltration is low in both wild-type and EphA4 knockout spinal cords following hemisection. Sections of hemisectioned spinal cord were double-labelled with the pan T-lymphocyte marker CD3 (red) and CD11b (green) to identify infiltrating T cells within injured tissue. Images include DAPI nuclear counterstain (blue). A low number of CD3+ T cells (indicated by arrowheads) sparsely populated the injury site in both wild-type and EphA4 knockout spinal cords at 2 (A, E), 4 (B, F), 14 (C, G) and 42 (D, H) days post-injury (d.p.i.). Scale bars: A, C, E, G, H = 50 µm; B, D, F = 20 µm.

Previous studies using the contusion model of SCI have identified peaks of T cell infiltration at approximately 2 and 6 weeks post-injury [27], [28]. Using the pan T-lymphocyte marker CD3, T cells were observed in the lesion sites of both genotypes as early as 2 days post-injury and also at 4, 14 and 42 days post-injury. (Fig. 2). CD3+ T cells were not observed in spared tissue or intact spinal cords (data not shown). Very low numbers of CD3+ T cells were observed in both horizontal and transverse sections at all post-injury timepoints (approximately one CD3+ cell or less per section), which prevented meaningful quantitation between genotypes or timepoints. There was no indication of increased T cell infiltration at 14 or 42 days post-injury.

### Area, Rostro-caudal Spread and Density of Macrophages/Activated Microglia in Injured Wild-type and EphA4 Knockout Spinal Cords

**Figure 3:**
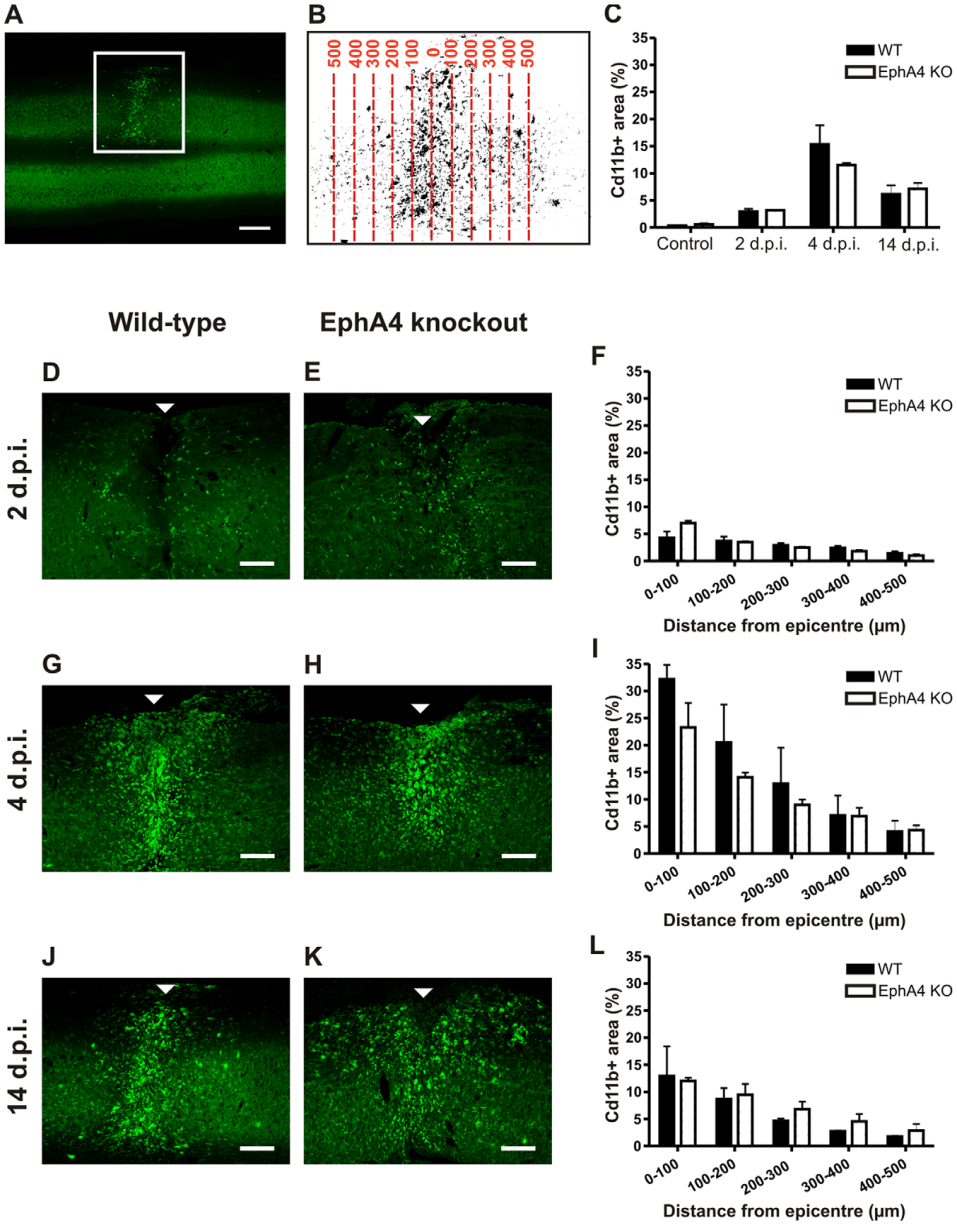
The rostro-caudal area and spread of macrophages/activated microglia does not differ between injured EphA4 knockout and wild-type spinal cords. Horizontal sections of spinal cord were immunostained with CD11b to label infiltrating macrophages and activated microglia. An example is shown in the low power image in A; the square surrounds the CD11b+ lesion on the left hand side of the spinal cord. CD11b immunoreactivity was converted to a positive area of staining by thresholding, and the proportional area of staining was analysed in 100 µm zones from the injury epicentre up to 500 µm rostrally and caudally (the example in B shows the injured left hand side of the spinal cord in A at higher power). Injured wild-type and EphA4 knockout spinal cords were analysed at 2 (D, E), 4 (G, H) and 14 (J, K) days post-injury (arrowheads indicate the injury epicentre). There was no significant difference between genotypes in the proportional CD11b+ area of any 100 µm rostro-caudal zone at 2 (F), 4 (I) or 14 (L) days post-injury. or within the total 1 mm rostro-caudal area in control spinal cords (C). Results show mean ± SEM of *n* = 3 wild-type and *n* = 2 EphA4 knockout controls; *n* = 3 per genotype at 2 and 4 days post-injury and *n* = 2 per genotype at 14 days. Scale bars: A = 500 µm; D, E, G, H, J, K = 200 µm. WT = wild-type, KO = knockout, d.p.i. = days post-injury.

The distribution of infiltrating macrophages and activated microglia in horizontal cryosections of injured wild-type and EphA4 knockout spinal cords was visualised with CD11b immunoreactivity at 2, 4 and 14 days post-injury and in uninjured control mice. The spread of cells was analysed by measuring the proportional area of CD11b immunoreactivity within 100 µm rostro-caudal zones up to 500 µm from the epicentre, and also by measuring the overall proportional area of CD11b+ immunoreactivity up to 500 µm from the injury epicentre in the rostral and caudal directions (i.e. a 1 mm rostro-caudal area) (Fig. 3 A,B). The average proportional area of CD11b+ staining in the 1 mm rostro-caudal area encompassing the injury was not significantly different between genotypes in uninjured spinal cords or between genotypes at any post-injury timepoint examined (Fig. 3C). At all timepoints and in both genotypes, some macrophages/activated microglia were present beyond 500 µm from the epicentre but in relatively low numbers.

**Figure 4:**
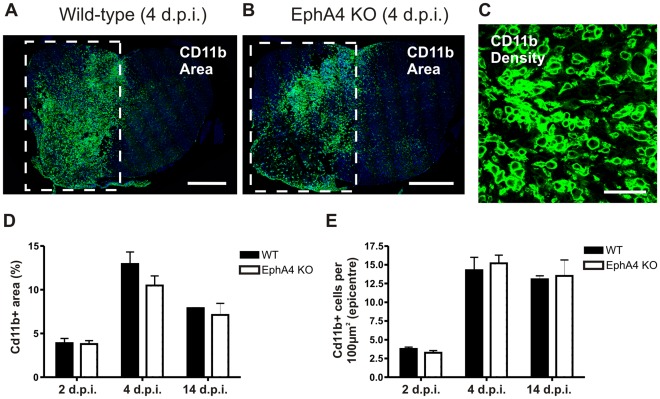
The density of macrophages/activated microglia in the lesion epicentre does not differ between injured wild-type and EphA4 knockout spinal cords. Transverse sections of hemisectioned spinal cord were immunostained with CD11b to label infiltrating macrophages and activated microglia. Examples from wild-type (A) and EphA4 knockout (B) spinal cords 4 days post-injury are shown; the square surrounds the CD11b+ lesions on the left hand side of the spinal cord. There was no difference between genotypes in the proportional area of CD11b immunoreactivity within the injury epicentre at 2, 4 or 14 days post-injury (D). Higher power images of CD11b+ staining (example in C) were used to determine the density of cells within the injury epicentre; no difference was seen between injured wild-type or EphA4 knockout mice at 2, 4 or 14 days post-injury (E). Images in A and B show DAPI nuclear counterstain. Results show mean ± SEM of *n* = 3 per genotype at 2 and 14 d.p.i. and *n* = 6 per genotype at 4 d.p.i. Scale bars: A, B = 500 µm; C = 50 µm. WT = wild-type, KO = knockout, d.p.i. = days post-injury.

The proportional area of CD11b+ staining within 100 µm rostral-caudal zones from the epicentre was analysed in the same sections. At each timepoint, there was a general trend for zones closer to the injury epicentre to have a higher proportion of tissue occupied by macrophages/activated microglia, and for the proportion to decrease with distance from the epicentre (Fig. 3F,I,L). At 2 days post-injury, rounded macrophages/activated microglia sparsely populated the necrotic tissue surrounding the hemisection in both genotypes (Fig. 3D,E,F). The largest area of CD11b staining was seen at 4 days post-injury, where there was also the greatest variation between samples, particularly close to the lesion centre. A large number of CD11b immunoreactive cells populated the injury site, compacted in the lesion centre (Fig. 3G,H,I). At 14 days, large numbers of CD11b+ macrophages/activated microglia continued to populate the hemisection site (Fig. 3J,K,L). Rather than the spherical shape observed at earlier post-injury timepoints, by 14 days post-injury a number of strongly CD11b immunoreactive cells had a less rounded morphology and short processes. There was no significant difference between genotypes in any individual rostro-caudal zone from the epicentre at all timepoints examined. This was confirmed by additional measurements in transverse sections through the lesion epicentre and at 100 µm rostral and caudal to the epicentre. (Fig. 4 A,B,D). CD11b+ cells within the lesion epicentre were also counted at higher power in transverse sections to confirm that the area of CD11b staining correlated with numbers of macrophages/activated microglia (Fig. 4C). There was no significant difference between genotypes in the density of CD11b+ cells at any post-injury timepoint examined (Fig. 4E).

### The Proportion of Arginase 1-positive Macrophages/Activated Microglia is Lower in EphA4 Knockout Compared to Wild-type Spinal Cords at 4 Days Post-injury

Arginase 1 is a marker of a macrophage subset, and Arginase 1 mRNA expression was three-fold lower in injured EphA4 knockout compared to wild-type spinal cords at 4 days post-injury. In sham-operated and naïve control spinal cords, Arginase 1 immunoreactivity was observed at low levels in motor neurons of the ventral horn. No Arginase 1 immunoreactivity was detected in any other cells in uninjured spinal cords of either genotype. Following injury, Arginase 1 immunoreactivity in surviving motor neurons exhibited no detectable change (data not shown).

**Figure 5:**
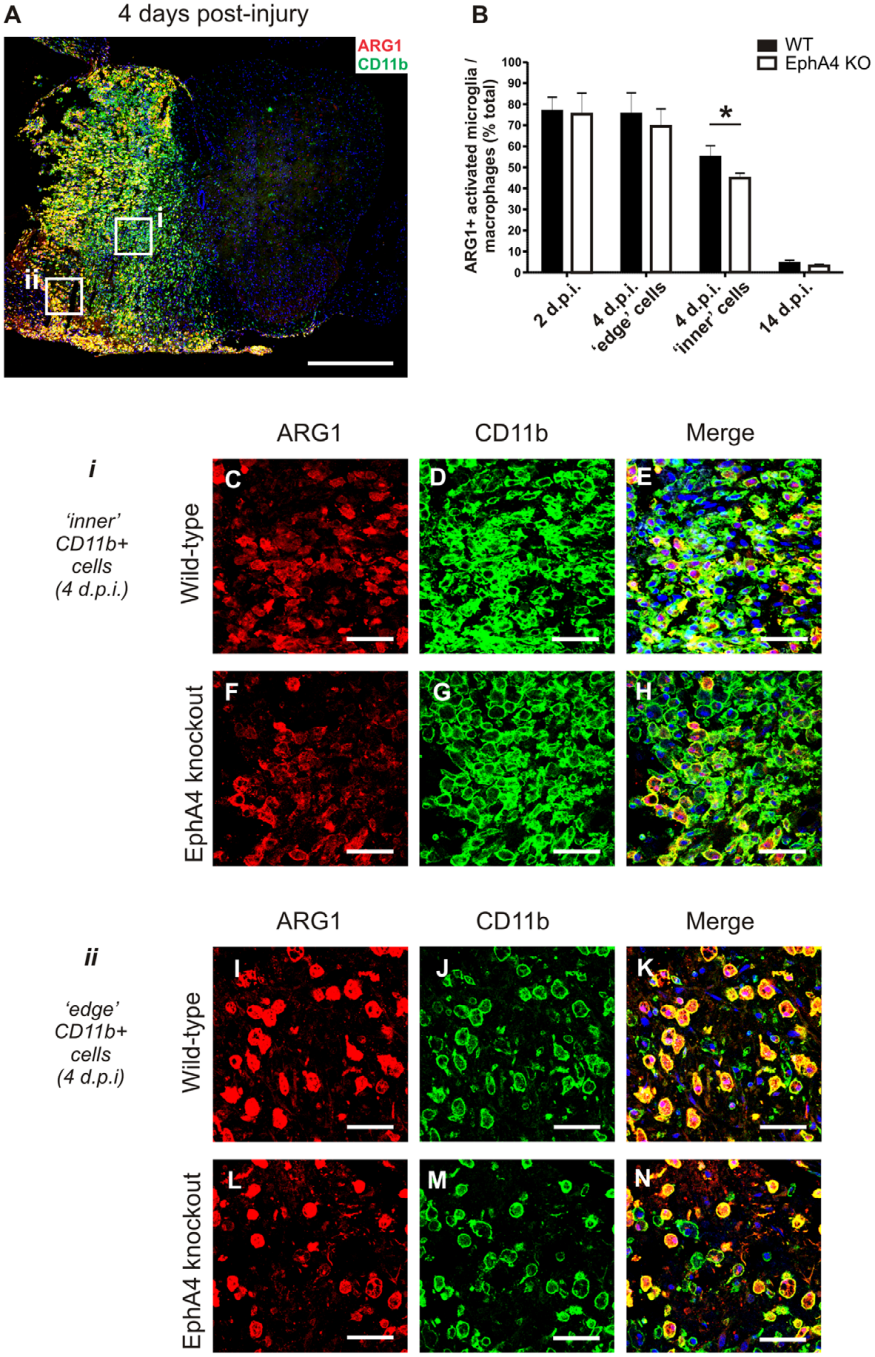
The proportion of Arginase 1-positive macrophages/activated microglia is lower in EphA4 knockout compared to wild-type spinal cords at 4 days post-injury. Transverse sections of hemisectioned spinal cord were double-labelled with Arginase 1 and CD11b to identify the subset of infiltrating macrophages/activated microglia with Arginase 1 immunoreactivity. A representative example from a wild-type spinal cord at 4 days post injury is shown in A, with positive staining on the injured left hand side. Cells with a foamy macrophage appearance located in areas of tissue necrosis (ii, coded ‘edge’ areas) were more likely to be strongly Arginase 1-immunoreactive than densely packed macrophages not surrounded by obvious areas of necrosis (i, coded ‘inner’ areas). There was a significantly lower proportion (*p = 0.0018, B) of Arginase 1-positive CD11b positive macrophages/activated microglia in densely packed ‘inner’ areas in EphA4 knockout (F-H) compared to wild-type (C-E) spinal cord lesions at 4 days but no difference in the proportion (B) of Arginase 1-positive macrophages/activated microglia in necrotic ‘edge’ areas in EphA4 knockout (L-N) and wild-type (I-K) lesions. There was no difference between genotypes in the proportion of Arginase 1-positive macrophages/activated microglia at 2 or 14 days post-injury (B), by which time the proportion of Arginase 1-positive macrophages/activated microglia had dropped dramatically. Merged images include DAPI nuclear counterstain. Results show mean ± SEM of *n* = 6 per genotype at 4 days and *n* = 3 per genotype at 2 days and 14 days post-injury Scale bars: A = 500 µm; C-E, F-H, I-K, L-N = 50 µm. ARG1 = Arginase 1, WT = wild-type, KO = knockout, d.p.i. = days post-injury.

In contrast, a large number of Arginase 1 and CD11b co-labelled cells were present in the lesion site of both genotypes following injury (Fig. 5A). At 4 days post-injury, Arginase 1 was strongly immunoreactive in many CD11b+ cells with a rounded morphology but was not detected in ramified CD11b+ cells (resting microglia). Qualitative analysis indicated that Arginase 1 positive macrophages/activated microglia were not randomly distributed throughout the lesion site; Arginase 1 immunoreactivity appeared to be stronger and present in a greater proportion of CD11b+ cells in areas of obvious necrosis or tissue loss, frequently in cells which resembled ‘foamy macrophages’. These areas tended to be on the periphery of the spinal cord in transverse sections, and were termed ‘edge’ areas (box ii in Fig. 5A). Arginase 1 immunoreactivity qualitatively appeared to be weaker and in a slightly lower proportion of CD11b+ cells which were densely packed together in the lesion centre. These areas tended to be located more centrally in the spinal cord in transverse sections, and were termed ‘inner’ areas (box i in Fig. 5A).

There was a significantly lower proportion (p = 0.0018, Fig. 5B) of Arginase 1/CD11b co-labelled cells in ‘inner’ areas in EphA4 knockout (Fig. 5C-E) compared to wild-type spinal cords (Fig. 5F–H) at 4 days post injury. In contrast, there was no significant difference in the proportion of Arginase 1 and CD11b co-labelled cells in ‘edge’ areas in EphA4 knockout and wild-type lesions (Fig. 5I-N). There was also no difference at 2 or 14 days post-injury between genotypes and no obvious difference in the distribution of Arginase 1 positive cells within the lesion site (Fig. 5B).

## Discussion

EphA4 knockout mice display axonal regrowth and functional recovery following spinal cord hemisection [6], however the altered mechanisms underlying this regeneration have not been fully elucidated. In order to identify altered processes which potentially contribute to this regeneration, gene expression profiling was conducted on injured and uninjured spinal cord tissue from EphA4 knockout and wild-type mice.

### Transcriptional Differences in the Uninjured EphA4 Knockout Spinal Cord

Comparison of spinal cords from sham-operated EphA4 knockout and wild-type mice reveals inherently modest (generally less than 2-fold) gene expression differences between the genotypes. A large proportion of significantly differentially expressed genes were related to the nucleus and the regulation of transcription. With application of the p-value, fold change filter and enrichment analysis described above, almost half of the differentially expressed genes were related to ‘intracellular organelle’ or similar categories, suggesting that fundamental differences may exist within cells of EphA4 knockout mice. However more work is required to determine whether, or how, these differences affect cellular function following injury.

### Inflammation-related genes are Differentially Expressed in EphA4 Knockout Spinal Cords Following Injury

Differential expression of a number of immune and inflammation-related genes following injury is of particular interest, as the post-traumatic neuroinflammatory response plays a role in both repair and increased secondary injury in the CNS. A number of inflammation-related genes were found to be differentially expressed between genotypes following injury, including genes related to the innate and adaptive immune responses. A group of genes were identified by hierarchical clustering as being upregulated after injury in both genotypes, but the increase was attenuated in EphA4 knockout compared to wild-type samples. These genes, including *Tlr6*, *Casp1*, *Cflar*, *Mapkapk2*, *Nupr1*, *Fcgr1*, *Ctla2a*, *Sla*, *Cd244* and *Cysltr1*, have diverse roles relating to the immune system and inflammation. For example, *Tlr6*, *Casp1* and *Cflar* are involved in the regulation of the innate immune response: *Tlr6* has been detected in mouse and human microglia and the TLR family is linked to microglial activation [29], [30]. Additional inflammation-related genes were identified in the list of genes that were differentially expressed between injured EphA4 knockout and wild-type samples including Arginase 1, the expression of which was approximately 3-fold lower in injured EphA4 knockout compared to wild-type samples. Arginase 1 is expressed in neurons of the spinal cord [31] and in a subset of macrophages including alternatively activated (M2) macrophages; the marked upregulation observed in injured samples in our experiment is likely due to an influx of Arginase 1 positive macrophages which are abundant in the days following rodent SCI [32].

The majority of immune/inflammation-related genes had an attenuated increase in expression in injured EphA4 knockout compared to wild-type samples, although the genes have diverse functions, reflected by few significant gene ontology groups related to specific inflammatory categories. Given that there was no apparent gross difference in the inflammatory response to spinal cord injury in the EphA4 knockout mice it suggested that specific components of the inflammatory response may be altered, as was the case for Arginase 1 at the specific timepoint of 4 days post-injury and even then only in a subset of macrophages.

### T Cell Infiltration in the Spinal Cord Hemisection Model

Studies of contusion SCI in C57/Bl6 mice indicate two peaks of T lymphocyte infiltration occur at approximately 2 and 6 weeks post-injury [27], [28], timepoints that were included in this study. Here we observed T cells in the lesion site as early as 2 days post-injury in both genotypes, but still only very low numbers at 14 and 42 days. As there are differences between contusion and hemisection models of SCI in the extent and pattern of tissue damage and inflammation [33], it is possible that T cell infiltration is simply low in this model. Alternatively, peak lymphocyte infiltration may occur at a different timepoint not examined here. Some differentially expressed genes from the microarray were related to the adaptive immune system and it should be noted that even low numbers of T cells in the lesion site may have an impact on the inflammatory response.

### Macrophage Infiltration and Microglial Activation

The number of macrophages/activated microglia, likely the most numerous immune cell type present in the acute phase of inflammation, was not attenuated in injured EphA4 knockout spinal cords. Altering macrophage properties without significantly decreasing their number may improve outcomes following CNS injury, as recently demonstrated in *Mapkapk2* knockout mice [34]. However, it is currently unclear what contribution, if any, the lower number of Arginase 1-positive macrophages is making to the axonal outgrowth observed in EphA4 knockout mice. Arginase 1 is highly expressed in a “pro-regenerative” subset of macrophages [35], [36], [37], known as alternatively activated macrophages, in response to parasitic infection via the STAT6 pathway. However it has recently been shown that they can also be detrimental [38], which may indicate tissue-specific variability in effects. The expression of other common alternatively activated macrophage markers, Chitinase 3-like 3 (*Ym1*), mannose receptor (*CD206*) and Found in inflammatory zone 1 (*Fizz1)*, were not significantly different between injured genotypes in our data. Similarly the microarray data did not provide evidence for a differential expression of inducible nitric oxide synthase, interleukin (IL)-4, IL-13 or the receptor IL4Rα, suggesting that other alternatively activated macrophage properties are not altered. Arginase 1 is also induced in classically activated (M1) macrophages in a STAT6 independent manner via the Toll–like receptor 2/MyD88 pathway following parasitic infection [39], [40]. Our data also did not identify any of the genes currently known to be involved in this pathway as differentially expressed although *Tlr6* expression was lower in injured EphA4 KO compared to injured WT spinal cords.

An Arginase 1 expressing macrophage population also expressing mannose receptor and *Fizz1,* characteristic of alternatively activated macrophages, was identified from the Immunological genome project website [41]. This population also expresses high levels (relative to other macrophage cell types) of a number of genes that we identified as having attenuated increased expression in our model (Table S1 and Fig. S4) (*Casp1, Tlr6,* RAB32, member of Ras oncogene family (*Rab32),* Baculoviral IAP repeat-containing 1f (*Birc1f),* Selenoprotein X 1 (*Sepx1),* Glucan (1,4-alpha-), branching enzyme 1 (*Gbe1),* Transmembrane protein 38b (*Tmem38b), Nupr1,* Paraoxonase 3 (*Pon3), Cysltr1 and Cd244)*. Expression of Arginase 1, *Nupr1*, *CD244* and *Fcgr1* at CNS injury sites is also strongly correlated in a murine cortical injury model (GDS2850) (Fig. S5) and a murine SCI contusion model (GDS2159) (Fig. S6 and S7), particularly at the early time points of 1 and 3 days post-injury. Together with our results these data support the hypothesis of an Arginase 1 expressing macrophage population that is present at an early time point after CNS injury. The pathway by which Arginase 1 is induced in macrophages within injured CNS tissue and the function of Arginase 1 at the injury site remains to be elucidated. However, previous studies have demonstrated Arginase 1 expression in macrophages results in lower levels of nitric oxide and subsequently reduced inflammation [42], [43].

Arginase 1-positive alternatively activated macrophages have been shown to be less neurotoxic and more pro-regenerative than classically activated macrophages *in vitro* [32] and it remains to be determined whether a lower proportion of alternatively activated macrophages could have any positive effects post-SCI. While the wound-healing properties of alternatively activated macrophages have been extensively characterised *in vitro* and in non-CNS tissue, this may not translate directly to the injured spinal cord. For example, one beneficial function of alternatively activated macrophages is collagen deposition [44] which may not promote regeneration in the injured CNS if the amount of fibrotic tissue is increased. Additionally, the ‘edge’ and ‘inner’ Arginase 1-positive macrophages may be contributing differently to the lesion environment. There was no difference between genotypes in the proportion of strongly Arginase 1-positive macrophages in ‘edge’ areas, which had the appearance of foamy macrophages and were likely to be phagocytosing tissue and myelin debris. Additionally, it is not certain that the relative proportion of activated microglia and haematogenous macrophages are equivalent in the lesions of wild-type and EphA4 knockout spinal cords, as immunohistochemistry could not discriminate between these cells. Determining this may be important as it has been suggested that ‘microglia-dominant inflammation’, with less involvement of macrophages, leads to better outcomes following SCI [45].

### Alterations in Macrophages/Microglia Versus Environmental Regulation of Phenotype

It is not clear whether the altered Arginase 1 expression is due to heterogeneity in the macrophages themselves or rather is indicative of altered factors in the lesion environment. It is possible that the altered expression of Arginase 1 in EphA4 knockout macrophages is indicative of other neuroinflammatory changes, whether or not it directly contributes to the axonal outgrowth observed. Macrophage phenotype is sensitive to factors in the surrounding environment and Arginase 1 expression differences may indicate altered levels of inflammatory cytokines in the lesion site, which peak soon after injury [46]. However, unlike the intact spinal cord, factors in the lesion environment do not appear to support an alternatively activated macrophage phenotype [32].

The altered proportion of Arginase 1-positive macrophages may be indicative of increased heterogeneity in the macrophage population. The juxtaposition of Arginase 1-positive and negative macrophages in densely populated regions of the lesion core, which are presumably exposed to similar factors in the environment, suggests that cues within the lesion may not account for all differences seen in macrophage phenotype. Factors which contribute to immune cell heterogeneity are still being elucidated [47]. Interpretation of the role of Arginase 1 expressing macrophages following CNS injury in our model is complicated by the fact that, while EphA4 has not been associated with myeloid or monocyte development, EphA4 protein has been localised to macrophages in human lung [48] and multiple sclerosis lesions [19] and other Eph receptors and ephrin ligands are expressed in monocytes and macrophages [10], [19]. EphA4 is also expressed in the murine Arginase 1 expressing macrophage population from the Immunological database described above [41] (Fig. S4).This raises the possibility that interaction of ephrin-expressing macrophages with EphA4-expressing astrocytes within the lesion environment [6], [8], [22] may regulate macrophage properties.

### Conclusions

Neuroinflammation is an important component of secondary injury and repair following SCI. While the microarray data indicates that a number of inflammation-related genes are differentially expressed in the injured spinal cord of EphA4 knockout mice, elucidating the contribution of these differences to regeneration may be challenging, especially if the cellular effects are subtle. Nonetheless, our results indicate that in addition to directly regulating axonal regeneration [6], [8] or astrocyte responsiveness [22], [23], EphA4 regulates other pathways that also contribute to repair of neural injury.

## Supporting Information

Figure S1 **Quality control of RNA and microarray samples.** A: A list of RNA samples used in the microarray and their RIN as indicated by Bioanalyser assessment. RNA samples had an RIN between 9 and 9.8 (out of 10) indicating all samples were of high quality. B: A 3 dimensional PCA plot mapping microarray samples based on their variation in genome-wide gene expression. Samples with similar profiles cluster closer together. As indicated by clustering of sham-operated samples of both genotypes (in blue) and SCI samples of both genotypes (in red), the largest variation was due to treatment and there were no biological or technical outliers. WT = wild-type; KO = knockout.
(TIF)

Figure S2 **Hierarchical clustering of differentially expressed genes generated by comparison of injured wild-type and EphA4 knockout samples (all genes labelled).** Hierarchical clustering identified subsets of differentially expressed genes with simr expression profiles across groups. KO = knockout.
(PDF)

Figure S3 **Histology of wild-type and EphA4 knockout spinal cords at multiple timepoints post-injury.** Transversely cryosectioned lumbar spinal cords from hemisectioned wild-type mice are shown at 2 (A), 4 (B), and 14 (C) days post-injury; the square surrounds the injured left hand side. Horizontally sectioned spinal cords from hemisectioned wild-type mice are shown at 2 (D), 4 (E), and 14 (F) days post-injury and EphA4 knockout spinal cords at the same post-injury timepoints are shown below (G-I). Full sections of spinal cord are shown in D and G while the left hand side is shown in E, F, H and I. Arrowheads indicate the injury site in D - I. Sections are stained with haematoxylin and eosin. Scale bars: A-C, D, G = 500 µm; E, F, H, I = 200 µm. d.p.i. = days post-injury.
(TIF)

Figure S4 **Gene expression profiles of selected genes of interest in murine immune cells obtained from Immunological genome database.** EphA4 is expressed in a small number of macrophage populations (A). One population, MF.Thio5.II-480hi.PC, co-expresses *Cd11b* (B) and also Arginase 1 (C) to a high level. This cell type also expresses *Nupr1* (D), *Fcgr1* (E) and *Cd244* (F).
(DOCX)

Figure S5 **Expression of** ***Arg1*** **,** ***Nupr1,*** **and** ***Fcgr1*** **have similar expression patterns in a murine cortical injury model.** Expression profile of selected genes from the GEO dataset: GDS2850– Brain trauma model: time course in *Mus musculus*. Analysis of brain at various time points up to 72 hours following lateral controlled cortical impact injury. Images are from the GEO website. The top line above each image details the experiment number and probe set number corresponding to the gene of interest. Along the Y axis is the relative gene expression level and along the X axis is the different tissue samples and time points included in the experiments. Note that the Y axis is a sliding scale that varies between each gene to allow subtle differences in values to be easily visualised, thus it is not appropriate to compare expression values between genes. Genes of interest presented are: *EphA4* (A), Arginase 1 (*Arg1*, B), *Nupr1* (C) *Fcgr1* (D) and *Cd244* (E).
(DOCX)

Figure S6 **Expression of** ***Arg1*** **,** ***Nupr1, CD244*** **and** ***Fcgr1*** **have similar expression patterns in a murine spinal cord injury model.** Expression profiles of selected genes from the GEO dataset: GDS2159– Spinal cord injury model: time course, *Mus musculus*. Analysis of the T8 spinal cord segment up to 28 days after moderate contusion injury. Gene expression at the site of impact compared to that at the adjacent rostral and caudal regions. Images are captured from the GEO website. The top line above each image details the experiment number and probe set number corresponding to the gene of interest. Along the Y axis is the relative gene expression level and along the X axis is the different tissue samples and time points included in the experiments. Note that the Y axis is a sliding scale that varies between each genes to allow subtle differences in values to be easily visualised, thus it is not appropriate to compare expression values between genes. Genes of interest presented are: *EphA4* (A), Arginase 1 (*Arg1*, B), *Nupr1* (C), *Fcgr1* (D), *CD244* (E), *Ctla2a* (F), *Sla* (G), *Casp1* (H), *Cflar* (I), *Mapkapk2* (J), *Tlr6* (K).
(DOCX)

Figure S7 **Correlation between gene expression of** ***Arg1*** **,** ***Nupr1*** **,** ***Fcgr1*** **and** ***Cd244*** **in CNS injury models over time.** Correlation plots for selected genes of interest showing positive expression correlations in both SCI and cortical CNS injury models (GDS2159 and GDS2850 respectively; see Fig S5 and S6) across multiple time points. Displayed are naïve control (blue points), sham injury control (green points) and injury samples (red points). The numbers within the data points indicates the injury time point in either hours (0.5 h, 4 h, 8 h) or days (1, 3, 7, 28) following injury. Positive correlation plots are shown for *Arg1* and *Nupr1* in SCI (Ai) and cortical injury (Aii); *Arg1* and *Fcgr1* in SCI (Bi) and cortical injury (Bii); *Arg1* and *CD244* in SCI (Ci) and cortical injury (Cii); and *Nupr1* and *Fcgr1* in SCI (Di) and cortical injury (Dii).
(DOCX)

Table S1 **Genes differentially expressed in injured wild-type and EphA4 knockout spinal cord samples.** Genes differentially expressed between genotypes following CNS injury, determined by a one-way ANOVA comparing injured groups only. Genes were considered differentially expressed between genotypes when *p*<0.01 and they had a fold change of ≥1.2. Genes are ordered as in Figure S2.
(DOC)
