# Supplementary figures and images for: Differential Gene Expression in the EphA4 Knockout Spinal Cord and Analysis of the Inflammatory Response Following Spinal Cord Injury
Source: PLoS One. 2012 May 22;7(5):e37635. doi: 10.1371/journal.pone.0037635 (PMC3358264; doi:10.1371/journal.pone.0037635)

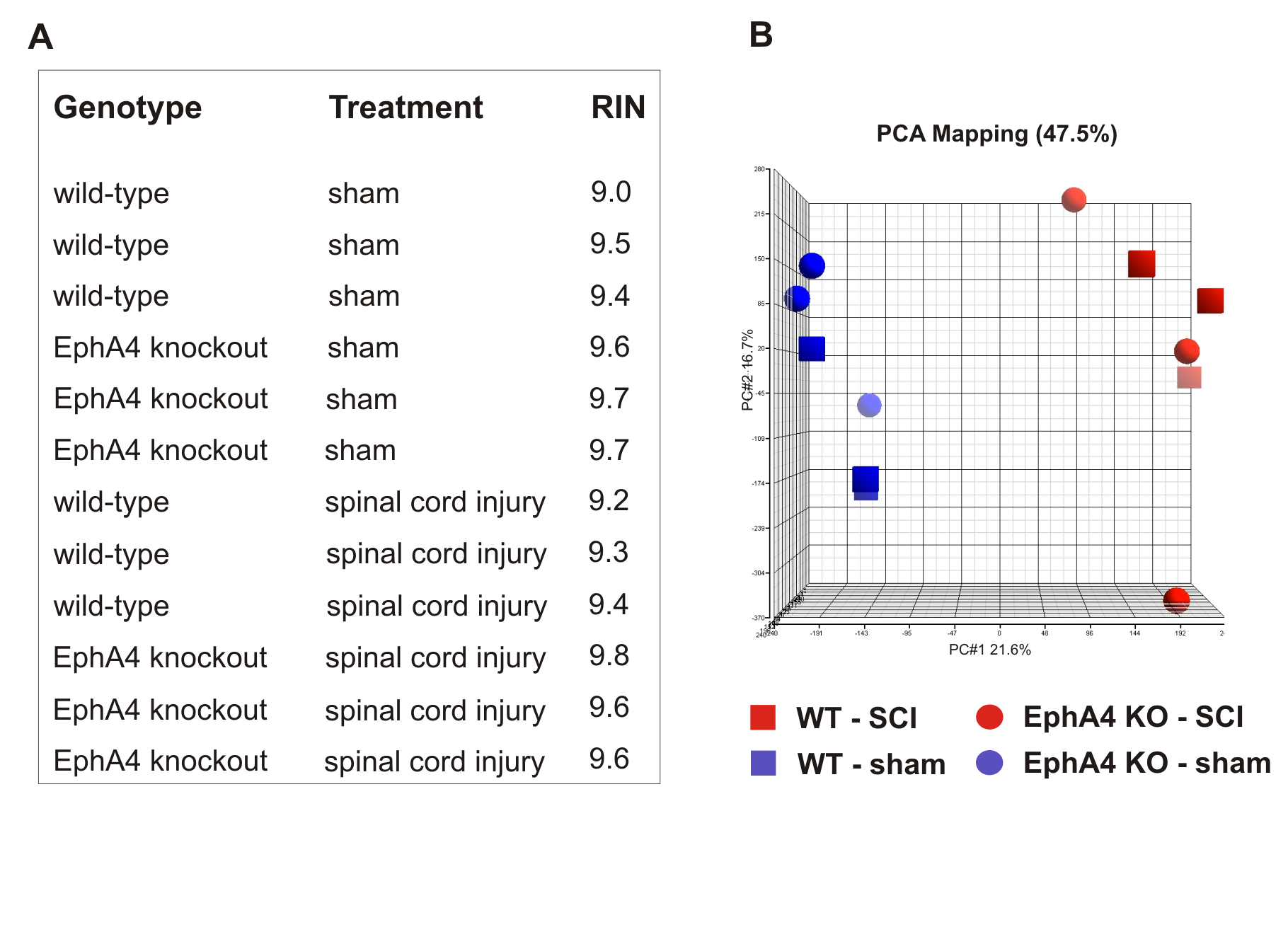

Supplement: Figure S1 — Quality control of RNA and microarray samples. A: A list of RNA samples used in the microarray and their RIN as indicated by Bioanalyser assessment. RNA samples had an RIN between 9 and 9.8 (out of 10) indicating all samples were of high quality. B: A 3 dimensional PCA plot mapping microarray samples based on their variation in genome-wide gene expression. Samples with similar profiles cluster closer together. As indicated by clustering of sham-operated samples of both genotypes (in blue) and SCI samples of both genotypes (in red), the largest variation was due to treatment and there were no biological or technical outliers. WT = wild-type; KO = knockout. (TIF) [file pone.0037635.s001.tif]

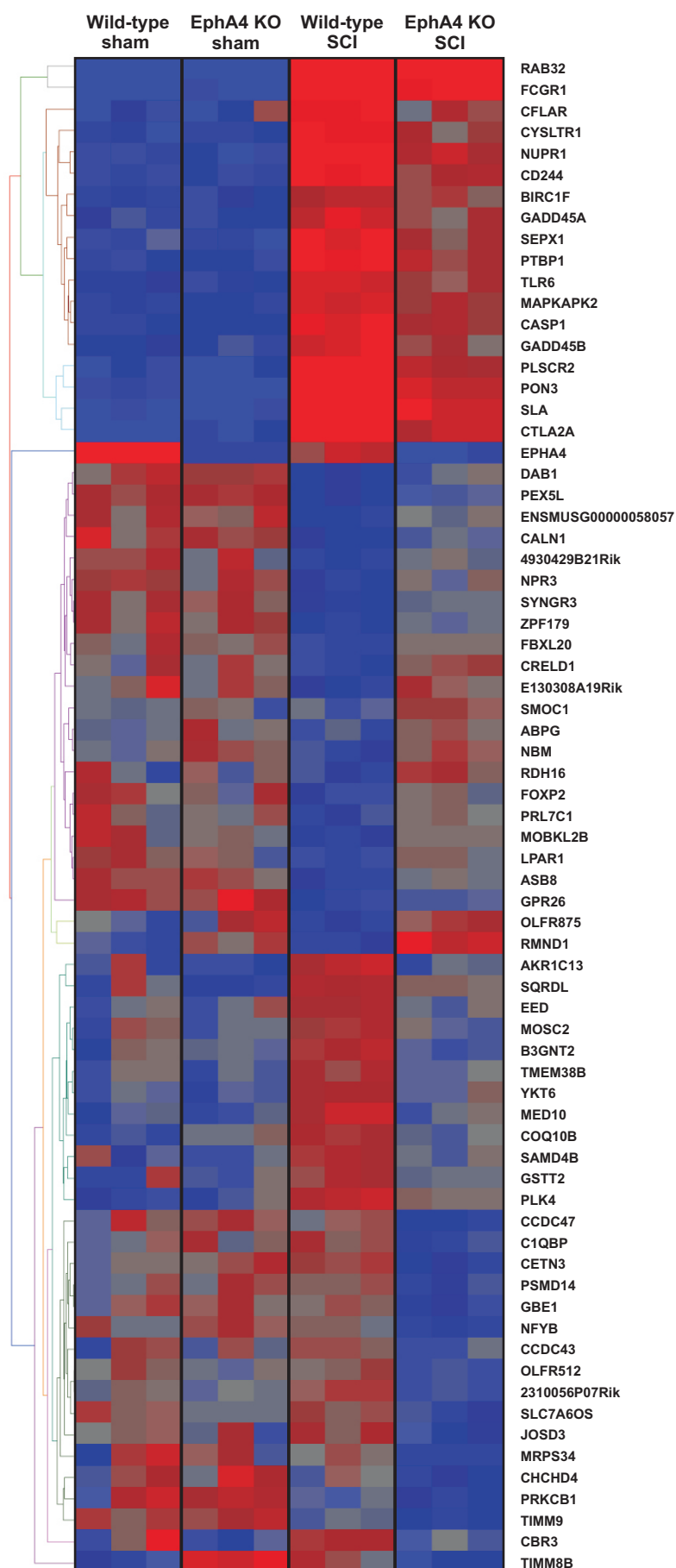

Low  High

Relative expression of a gene between groups

Supplement: Figure S2 — Hierarchical clustering of differentially expressed genes generated by comparison of injured wild-type and EphA4 knockout samples (all genes labelled). Hierarchical clustering identified subsets of differentially expressed genes with simr expression profiles across groups. KO = knockout. (PDF) [file pone.0037635.s002.pdf]

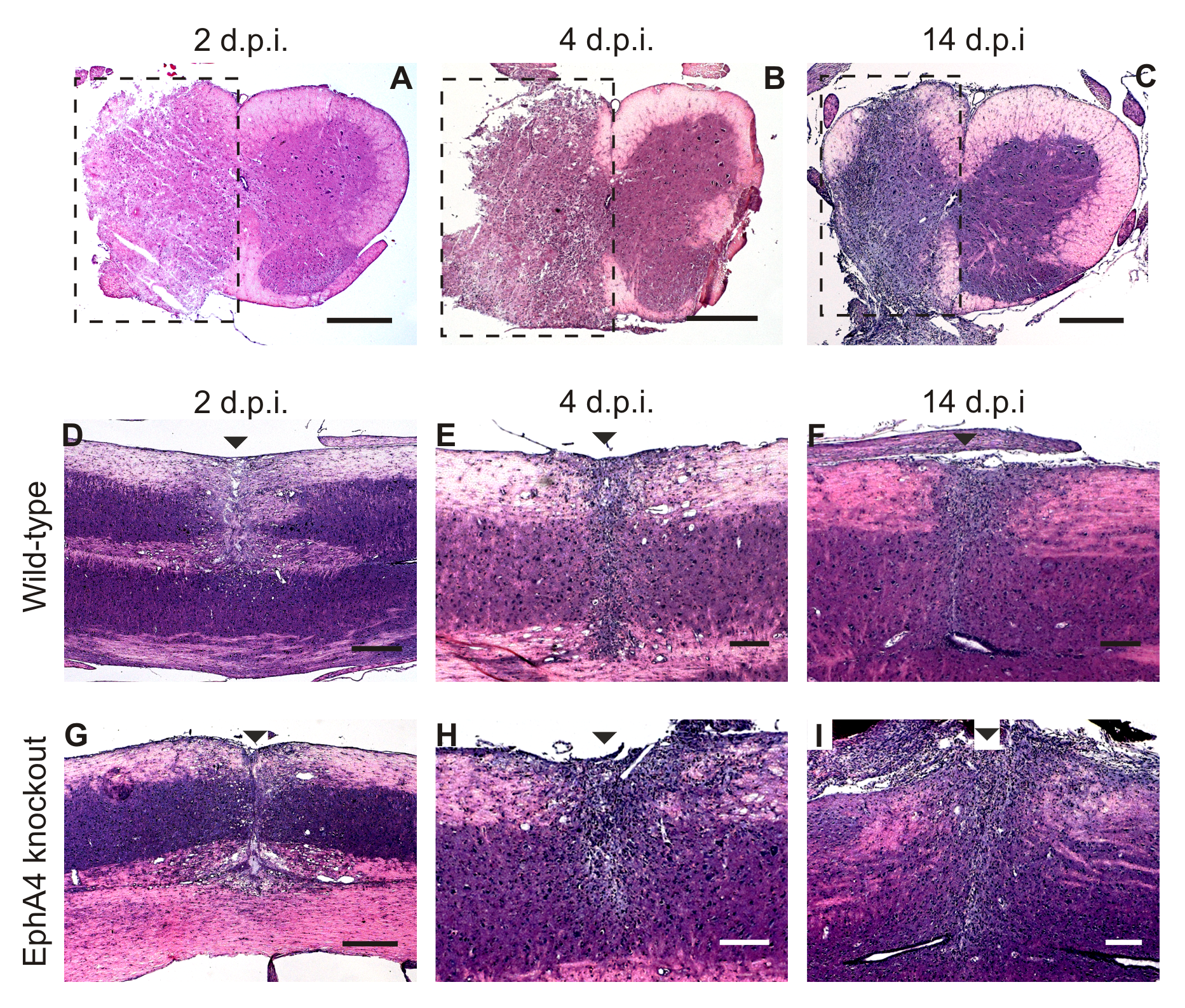

Supplement: Figure S3 — Histology of wild-type and EphA4 knockout spinal cords at multiple timepoints post-injury. Transversely cryosectioned lumbar spinal cords from hemisectioned wild-type mice are shown at 2 (A), 4 (B), and 14 (C) days post-injury; the square surrounds the injured left hand side. Horizontally sectioned spinal cords from hemisectioned wild-type mice are shown at 2 (D), 4 (E), and 14 (F) days post-injury and EphA4 knockout spinal cords at the same post-injury timepoints are shown below (G-I). Full sections of spinal cord are shown in D and G while the left hand side is shown in E, F, H and I. Arrowheads indicate the injury site in D - I. Sections are stained with haematoxylin and eosin. Scale bars: A-C, D, G = 500 µm; E, F, H, I = 200 µm. d.p.i. = days post-injury. (TIF) [file pone.0037635.s003.tif]
