# Supplementary material for: Differential Gene Expression in the EphA4 Knockout Spinal Cord and Analysis of the Inflammatory Response Following Spinal Cord Injury
Source: PLoS One. 2012 May 22;7(5):e37635. doi: 10.1371/journal.pone.0037635 (PMC3358264; doi:10.1371/journal.pone.0037635)
Supplement: Figure S4 — Gene expression profiles of selected genes of interest in murine immune cells obtained from Immunological genome database. EphA4 is expressed in a small number of macrophage populations (A). One population, MF.Thio5.II-480hi.PC, co-expresses Cd11b (B) and also Arginase 1 (C) to a high level. This cell type also expresses Nupr1 (D), Fcgr1 (E) and Cd244 (F). (DOCX) [file pone.0037635.s004.docx]

**Supplementary Figure S4: Gene expression profiles of selected genes of interest in murine immune cells obtained from** [**Immunological genome project**](http://www.immgen.org/index_content.html) **website.**

1. EphA4

1. CD11b

1. Arginase 1

1. Nupr1

1. Fcgr1

1. Cd244
