# Supplementary material for: Differential Gene Expression in the EphA4 Knockout Spinal Cord and Analysis of the Inflammatory Response Following Spinal Cord Injury
Source: PLoS One. 2012 May 22;7(5):e37635. doi: 10.1371/journal.pone.0037635 (PMC3358264; doi:10.1371/journal.pone.0037635)

**Supplementary Figure S5: Expression of *Arg1*, *Nupr1,* and *Fcgr1* have similar expression patterns in a murine cortical injury model (**[GDS2850](http://www.ncbi.nlm.nih.gov/sites/GDSbrowser?acc=GDS2850)).

A. **Eph receptor A4 (EphA4)**


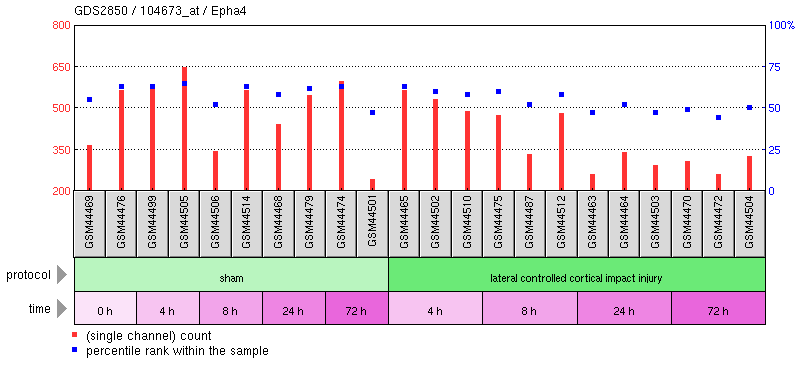


B. **Arginase 1 (Arg1)**


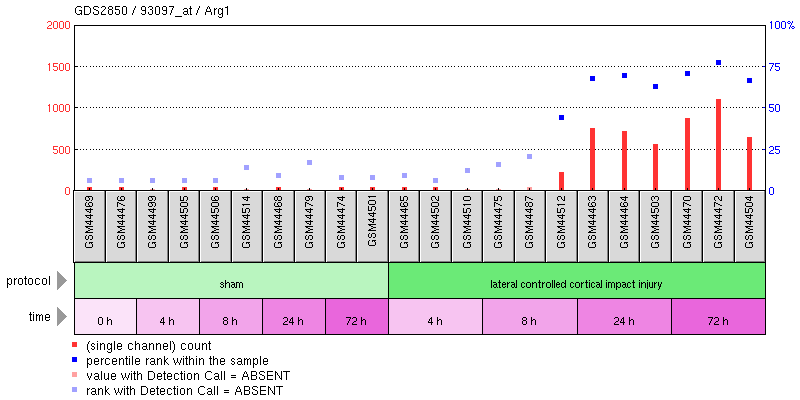


C. **Nupr1**


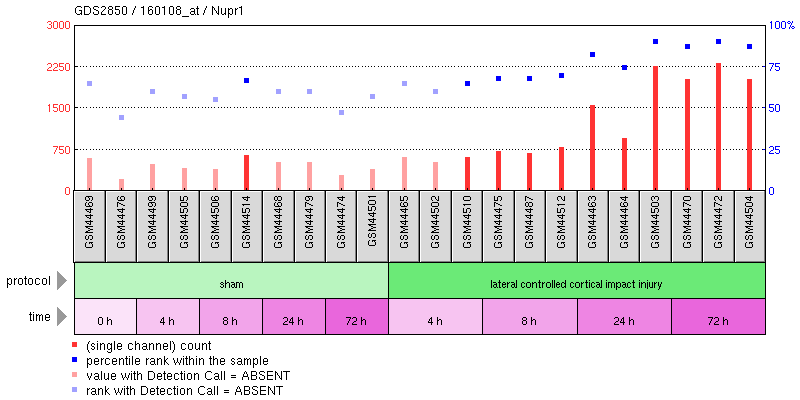


D. **FCGR1**


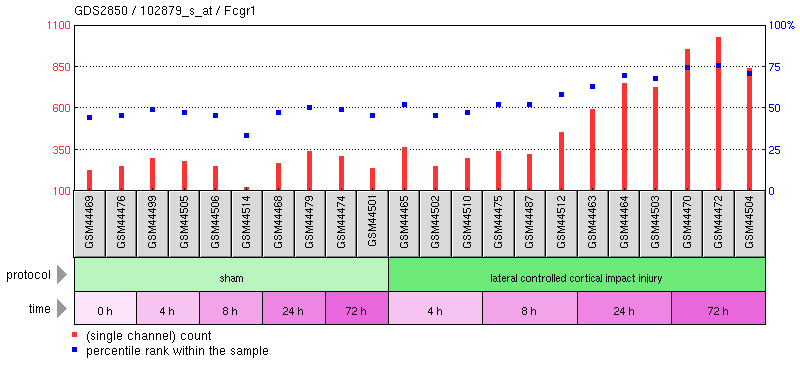


1. **CD244**


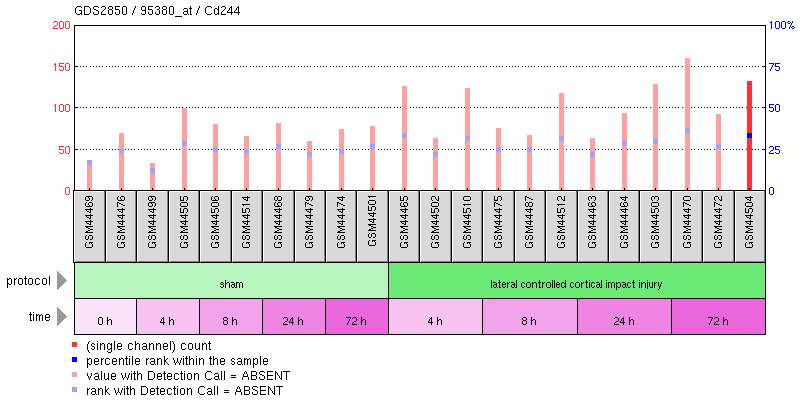

Supplement: Figure S5 — Expression of Arg1 , Nupr1, and Fcgr1 have similar expression patterns in a murine cortical injury model. Expression profile of selected genes from the GEO dataset: GDS2850– Brain trauma model: time course in Mus musculus. Analysis of brain at various time points up to 72 hours following lateral controlled cortical impact injury. Images are from the GEO website. The top line above each image details the experiment number and probe set number corresponding to the gene of interest. Along the Y axis is the relative gene expression level and along the X axis is the different tissue samples and time points included in the experiments. Note that the Y axis is a sliding scale that varies between each gene to allow subtle differences in values to be easily visualised, thus it is not appropriate to compare expression values between genes. Genes of interest presented are: EphA4 (A), Arginase 1 (Arg1, B), Nupr1 (C) Fcgr1 (D) and Cd244 (E). (DOCX) [file pone.0037635.s005.docx]
