# Supplementary material for: Differential Gene Expression in the EphA4 Knockout Spinal Cord and Analysis of the Inflammatory Response Following Spinal Cord Injury
Source: PLoS One. 2012 May 22;7(5):e37635. doi: 10.1371/journal.pone.0037635 (PMC3358264; doi:10.1371/journal.pone.0037635)

**Supplementary Figure S6: Expression of *Arg1*, *Nupr1, CD244* and *Fcgr1* have similar expression patterns in a murine spinal cord injury model (**[**GDS2159**](http://www.ncbi.nlm.nih.gov/sites/GDSbrowser?acc=GDS2159)**).**

A. Eph receptor A4 (Epha4)


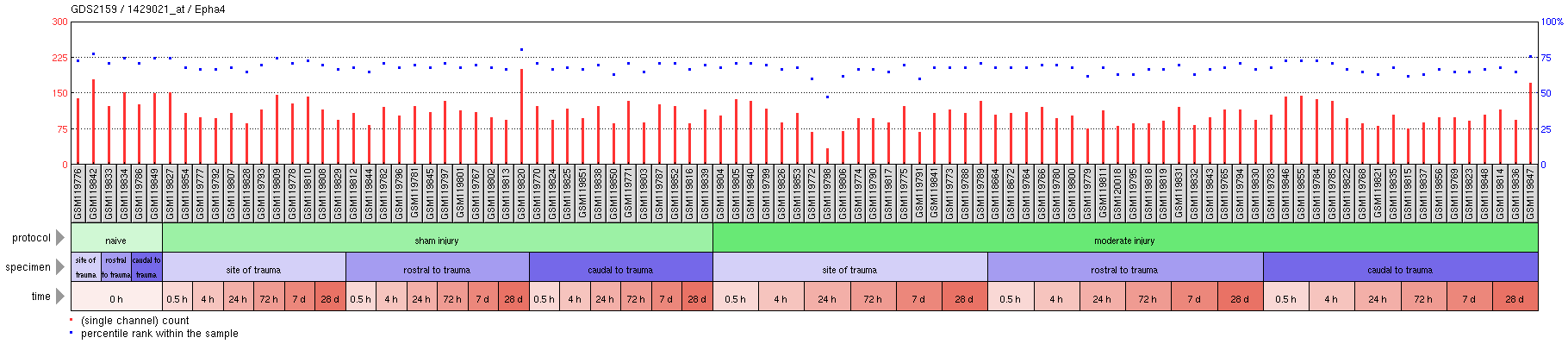


B. Arginase 1 (Arg1)


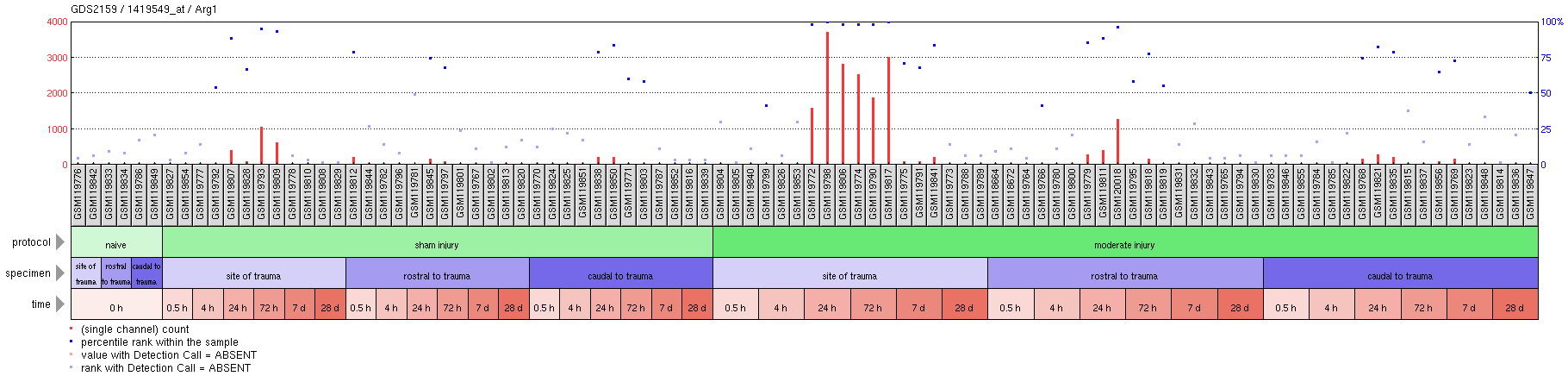


C. Nuclear protein 1 (Nupr1)


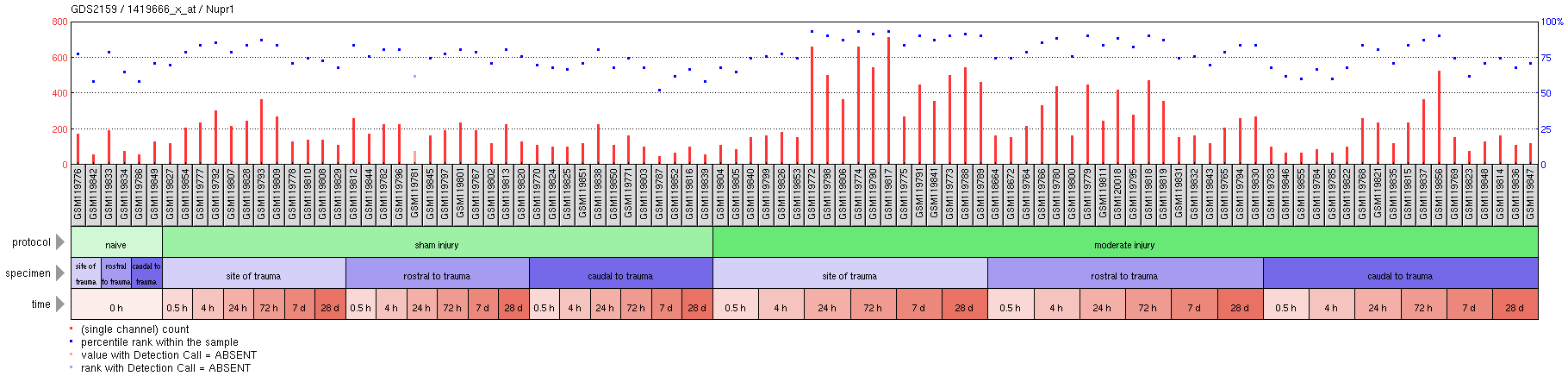


D. Fc receptor, IgG, high affinity 1 (Fcgr1)


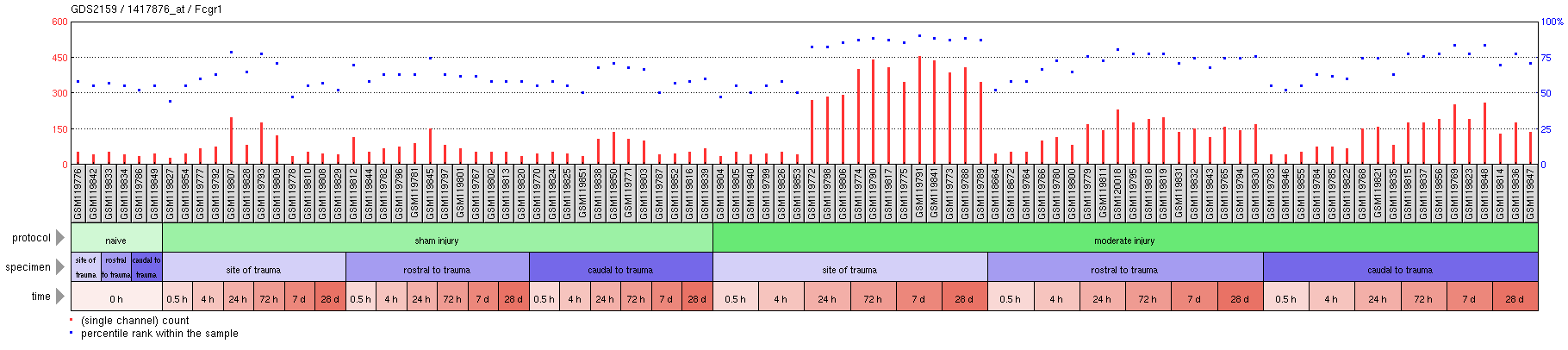


E. CD244 natural killer cell receptor 2B4 (CD244)


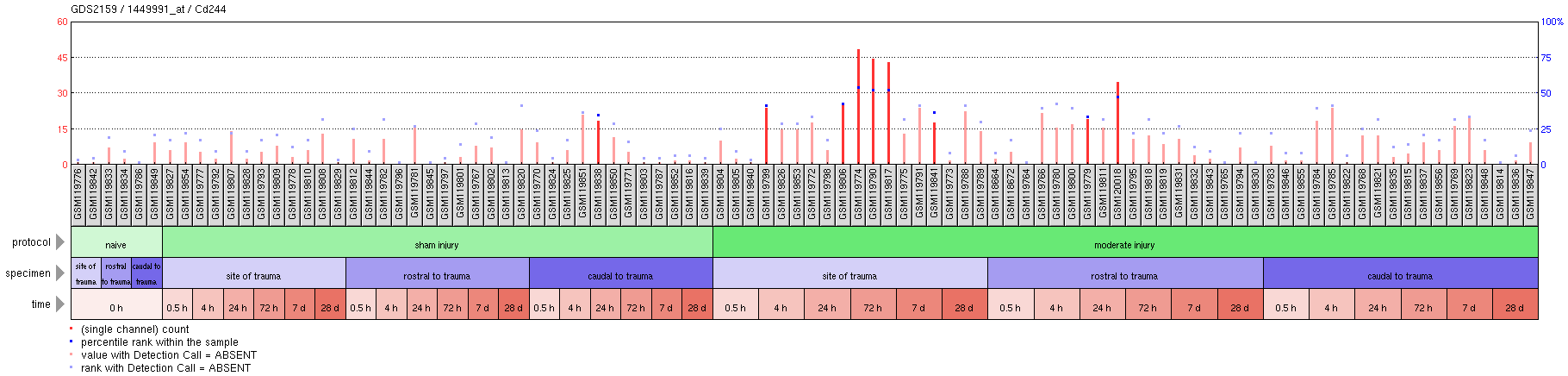


F. Cytotoxic T lymphocyte-associated protein 2 alpha (Ctla2a)


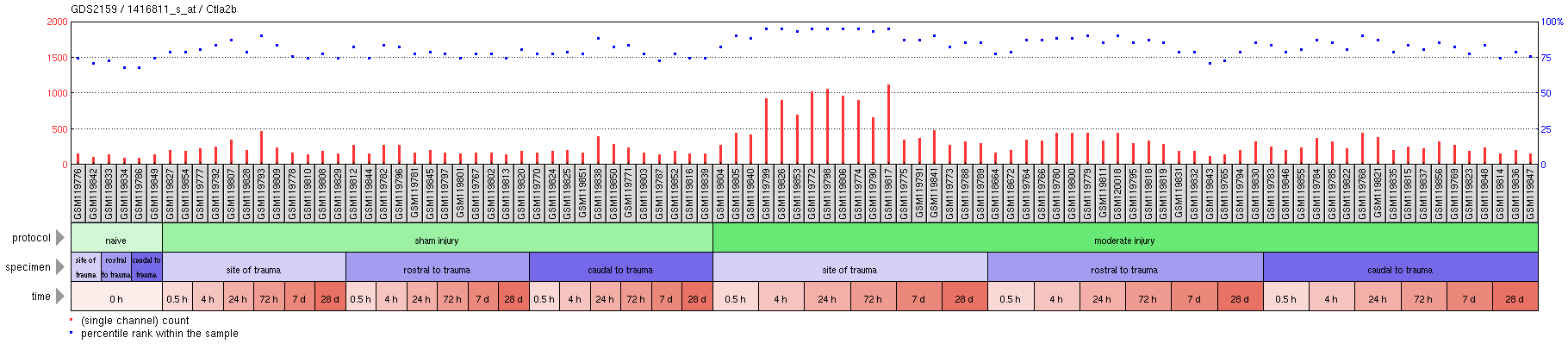


G. Src-like adaptor (Sla)


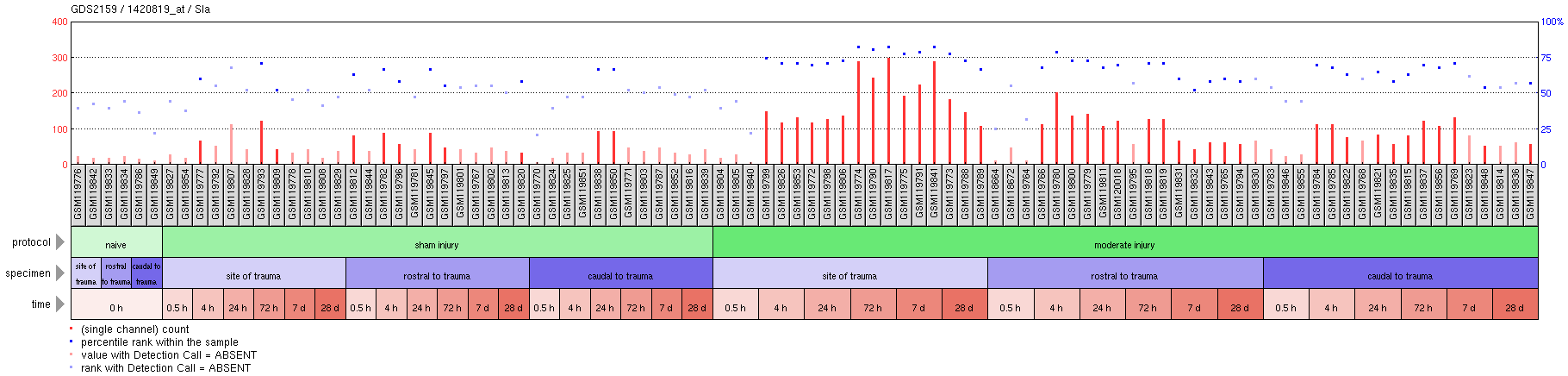


H. Caspase 1 (Casp1)


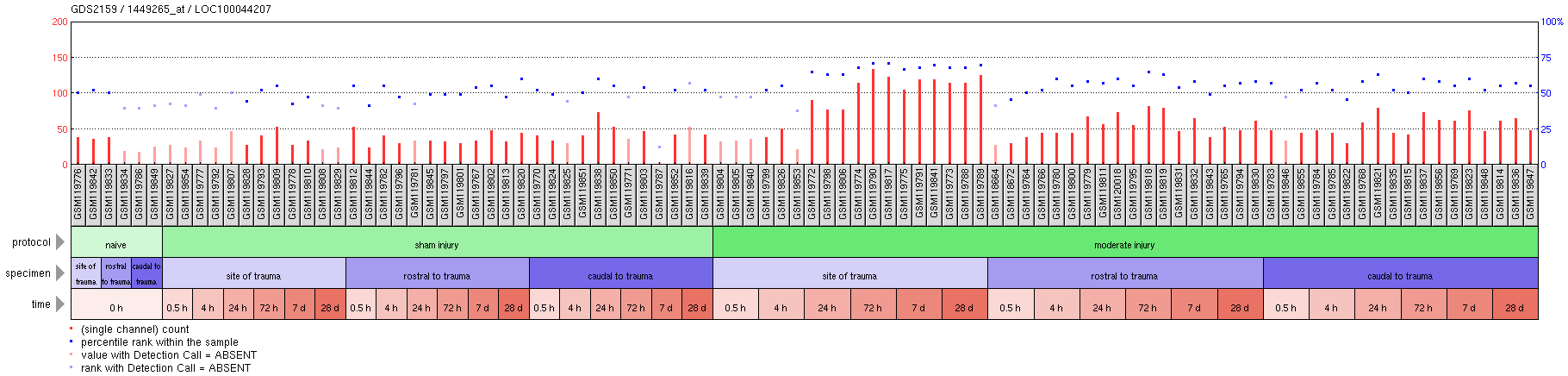


I. CASP8 and FADD-like apoptosis regulator (Cflar)


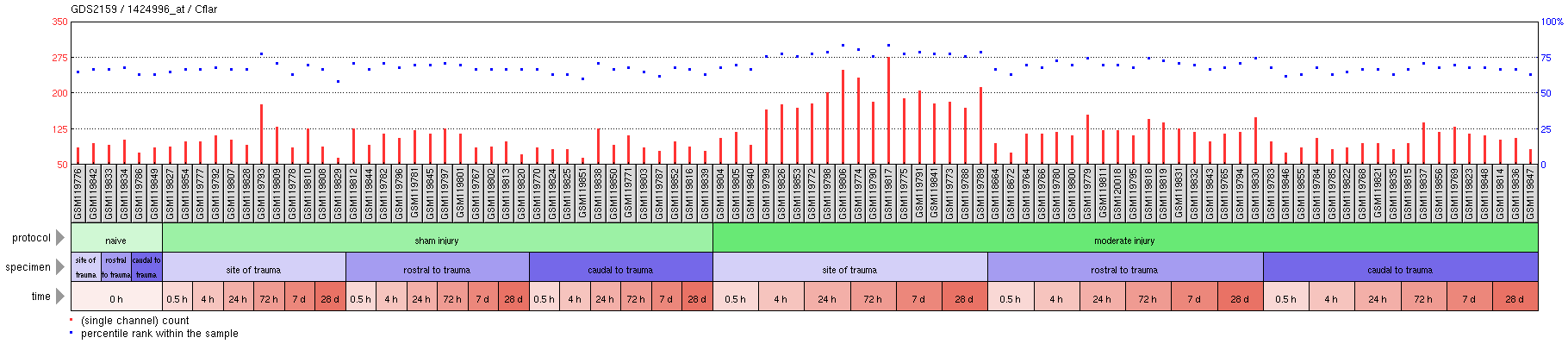


J. MAP kinase-activated protein kinase 2 (Mapkapk2)


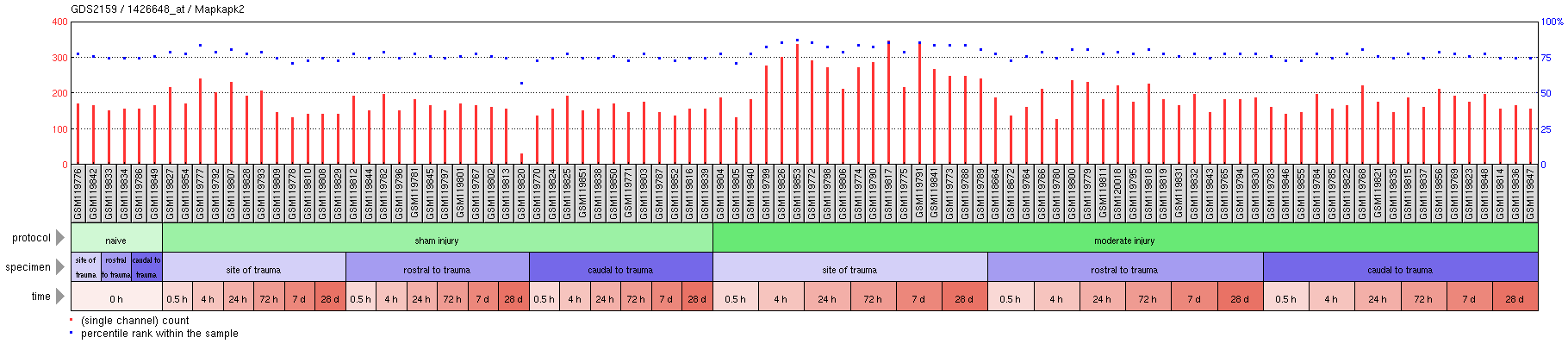


K. Toll-like receptor 6 (Tlr6)


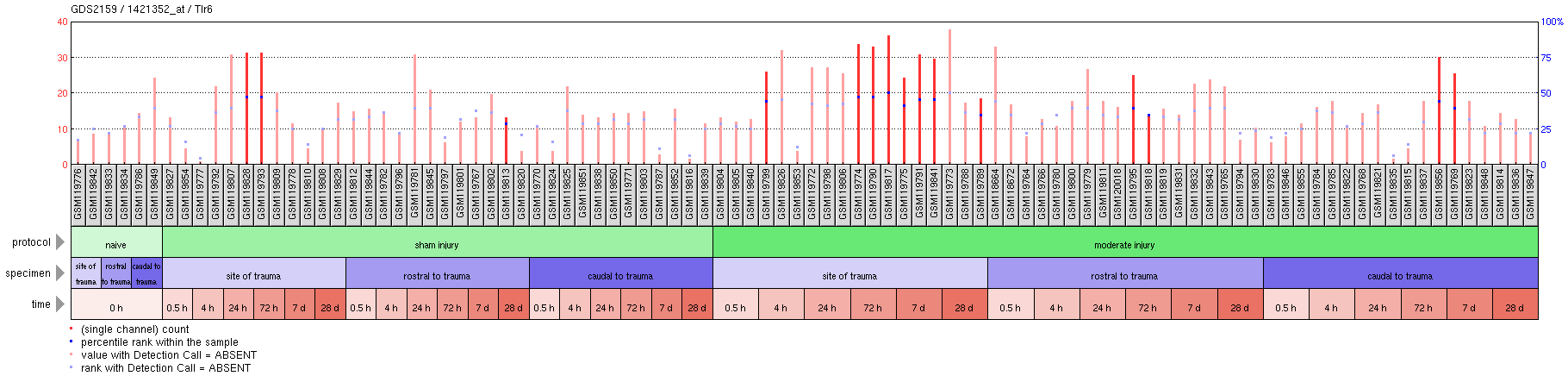

Supplement: Figure S6 — Expression of Arg1 , Nupr1, CD244 and Fcgr1 have similar expression patterns in a murine spinal cord injury model. Expression profiles of selected genes from the GEO dataset: GDS2159– Spinal cord injury model: time course, Mus musculus. Analysis of the T8 spinal cord segment up to 28 days after moderate contusion injury. Gene expression at the site of impact compared to that at the adjacent rostral and caudal regions. Images are captured from the GEO website. The top line above each image details the experiment number and probe set number corresponding to the gene of interest. Along the Y axis is the relative gene expression level and along the X axis is the different tissue samples and time points included in the experiments. Note that the Y axis is a sliding scale that varies between each genes to allow subtle differences in values to be easily visualised, thus it is not appropriate to compare expression values between genes. Genes of interest presented are: EphA4 (A), Arginase 1 (Arg1, B), Nupr1 (C), Fcgr1 (D), CD244 (E), Ctla2a (F), Sla (G), Casp1 (H), Cflar (I), Mapkapk2 (J), Tlr6 (K). (DOCX) [file pone.0037635.s006.docx]
