# Supplementary material for: Differential Gene Expression in the EphA4 Knockout Spinal Cord and Analysis of the Inflammatory Response Following Spinal Cord Injury
Source: PLoS One. 2012 May 22;7(5):e37635. doi: 10.1371/journal.pone.0037635 (PMC3358264; doi:10.1371/journal.pone.0037635)

**Supplementary Figure S7: Correlation between gene expression of *Arg1* , *Nupr1*, *Fcgr1* and *Cd244* in CNS injury models over time.**

Ai Aii


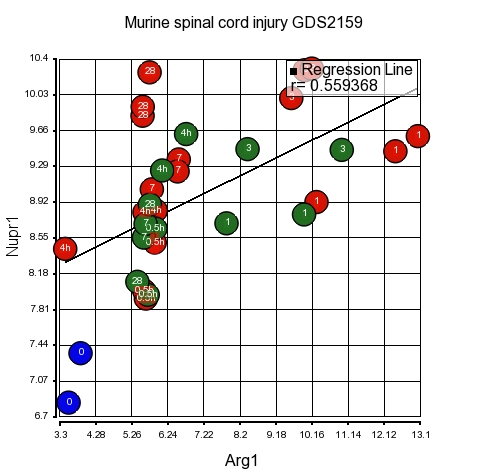

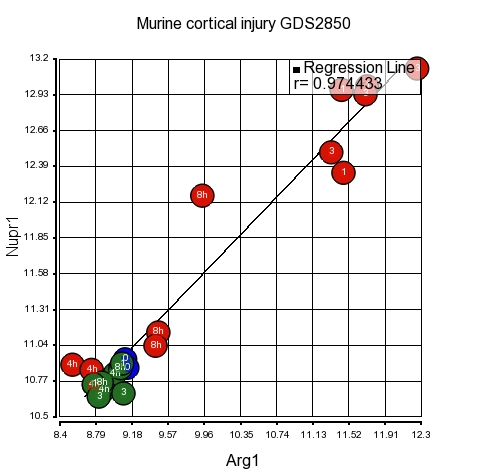


Bi Bii


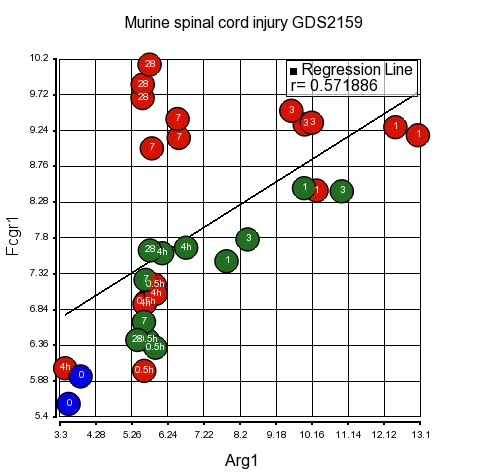

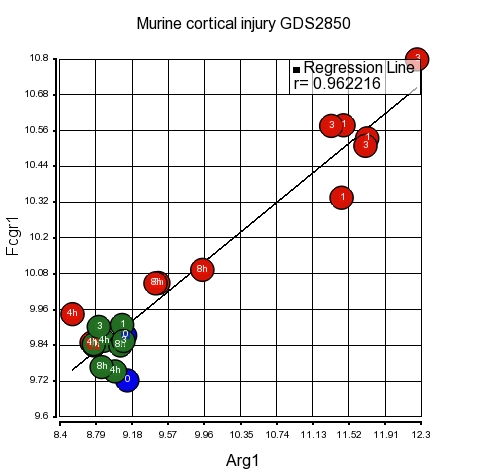


Ci Cii


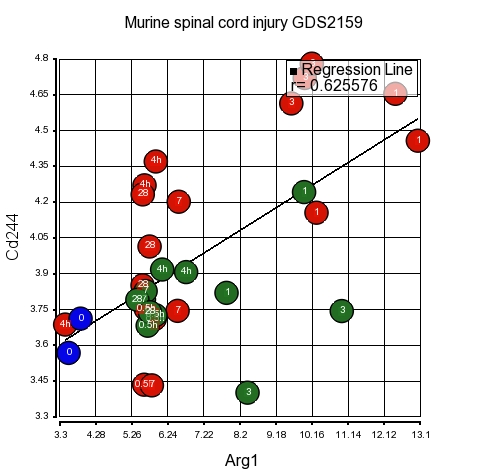

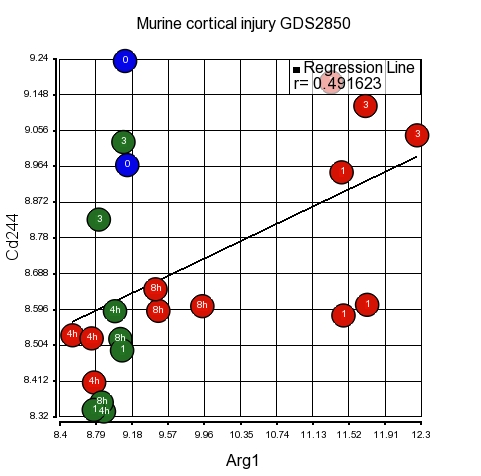


Di Dii


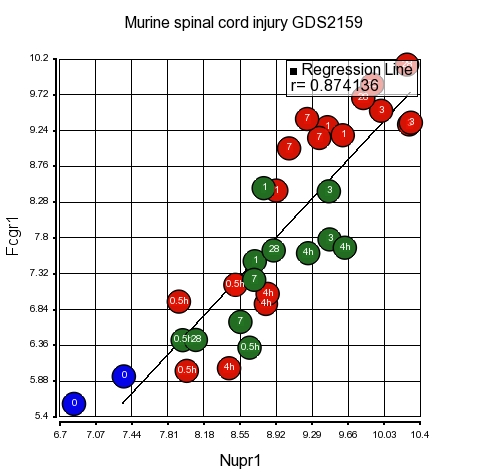

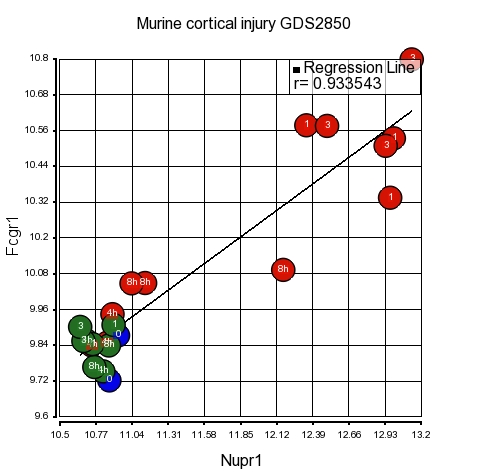

Supplement: Figure S7 — Correlation between gene expression of Arg1 , Nupr1 , Fcgr1 and Cd244 in CNS injury models over time. Correlation plots for selected genes of interest showing positive expression correlations in both SCI and cortical CNS injury models (GDS2159 and GDS2850 respectively; see Fig S5 and S6) across multiple time points. Displayed are naïve control (blue points), sham injury control (green points) and injury samples (red points). The numbers within the data points indicates the injury time point in either hours (0.5 h, 4 h, 8 h) or days (1, 3, 7, 28) following injury. Positive correlation plots are shown for Arg1 and Nupr1 in SCI (Ai) and cortical injury (Aii); Arg1 and Fcgr1 in SCI (Bi) and cortical injury (Bii); Arg1 and CD244 in SCI (Ci) and cortical injury (Cii); and Nupr1 and Fcgr1 in SCI (Di) and cortical injury (Dii). (DOCX) [file pone.0037635.s007.docx]
