# Supplementary material for: Differential Gene Expression in the EphA4 Knockout Spinal Cord and Analysis of the Inflammatory Response Following Spinal Cord Injury
Source: PLoS One. 2012 May 22;7(5):e37635. doi: 10.1371/journal.pone.0037635 (PMC3358264; doi:10.1371/journal.pone.0037635)
Supplement: Table S1 — Genes differentially expressed in injured wild-type and EphA4 knockout spinal cord samples. Genes differentially expressed between genotypes following CNS injury, determined by a one-way ANOVA comparing injured groups only. Genes were considered differentially expressed between genotypes when p<0.01 and they had a fold change of ≥1.2. Genes are ordered as in Figure S2. (DOC) [file pone.0037635.s008.doc]

**Supplementary Table S1: Genes differentially expressed in injured wild-type and EphA4 knockout spinal cord samples**

(p<0.01, fold change ≥1.2)

| **Cluster** | **Gene name** | **Gene symbol** | **RefSeq** | **p value** | **Fold change (to WT)** |
| --- | --- | --- | --- | --- | --- |
| A | RAB32, member RAS oncogene family | Rab32 | NM_026405 | 0.001056 | -1.45 |
| A | Fc receptor, IgG, high affinity I | Fcgr1 | NM_010186 | 0.004989 | -1.23 |
| A | CASP8 and FADD-like apoptosis regulator | Cflar | NM_207653 | 0.007871 | -1.56 |
| A | Cysteinyl leukotriene receptor 1 | Cysltr1 | NM_021476 | 0.009953 | -1.53 |
| A | Nuclear protein 1 | Nupr1 | NM_019738 | 0.007254 | -1.34 |
| A | CD244 natural killer cell receptor 2B4 | Cd244 | NM_018729 | 0.006755 | -1.43 |
| A | Baculoviral IAP repeat-containing 1f | Birc1f | NM_010871 | 0.004872 | -1.23 |
| A | Growth arrest and DNA-damage-inducible 45 alpha | Gadd45a | NM_007836 | 0.008497 | -1.33 |
| A | Selenoprotein X 1 | Sepx1 | NM_013759 | 0.005546 | -1.44 |
| A | Polypyrimidine tract binding protein 1 | Ptbp1 | NM_001077363 | 0.009336 | -1.39 |
| A | Toll-like receptor 6 | Tlr6 | NM_011604 | 0.002092 | -1.34 |
| A | MAP kinase-activated protein kinase 2 | Mapkapk2 | NM_008551 | 0.003842 | -1.26 |
| A | Caspase 1 | Casp1 | NM_009807 | 0.000840 | -1.37 |
| A | Growth arrest and DNA-damage-inducible 45 beta | Gadd45b | NM_008655 | 0.009562 | -1.42 |
| A | Phospholipid scramblase 2 | Plscr2 | NM_008880 | 0.006992 | -1.66 |
| A | Paraoxonase 3 | Pon3 | NM_173006 | 0.009206 | -1.35 |
| A | Src-like adaptor | Sla | NM_001029841 | 0.008711 | -1.46 |
| A | Cytotoxic T lymphocyte-associated protein 2 alpha | Ctla2a | NM_007796 | 0.008642 | -1.52 |
|  | Eph receptor A4 | Epha4 | NM_007936 | 0.001481 | -2.20 |
| B | Disabled homolog 1 (Drosophila) | Dab1 | NM_010014 | 0.009718 | 1.29 |
| B | Peroxisomal biogenesis factor 5-like | Pex5l | NM_021483 | 0.000824 | 1.23 |
| B | Predicted gene, ENSMUSG00000058057 | ENSMUSG00000058057 | NM_001081471 | 0.005115 | 1.33 |
| B | Calneuron 1 | Caln1 | NM_181045 | 0.001020 | 1.27 |
| B | RIKEN cDNA 4930429B21 gene | 4930429B21Rik | ENSMUST00000057768 | 0.006589 | 1.23 |
| B | Natriuretic peptide receptor 3 | Npr3 | NM_008728 | 0.008008 | 1.29 |
| B | Synaptogyrin 3 | Syngr3 | NM_011522 | 0.000763 | 1.24 |
| B | Zinc finger protein 179 | Zfp179 | NM_009548 | 0.006037 | 1.24 |
| B | F-box and leucine-rich repeat protein 20 | Fbxl20 | NM_028149 | 0.000437 | 1.21 |
| B | Cysteine-rich with EGF-like domains 1 | Creld1 | NM_133930 | 0.003754 | 1.37 |
| B | RIKEN cDNA E130308A19 gene | E130308A19Rik | NM_153158 | 0.001501 | 1.51 |
| B | SPARC related modular calcium binding 1 | Smoc1 | NM_022316 | 0.005291 | 1.22 |
| B | Androgen binding protein gamma | Abpg | NM_178308 | 0.009593 | 1.23 |
| B | Neuromedin B | Nmb | NM_026523 | 0.008867 | 1.33 |
| B | Retinol dehydrogenase 16 | Rdh16 | NM_009040 | 0.006259 | 1.36 |
| B | Forkhead box P2 | Foxp2 | NM_053242 | 0.005294 | 1.21 |
| B | Prolactin family 7, subfamily c, member 1 | Prl7c1 | NM_026206 | 0.009319 | 1.21 |
| B | MOB1, Mps One Binder kinase activator-like 2B (yeast) | Mobkl2b | NM_178061 | 0.000164 | 1.27 |
| B | Lysophosphatidic acid receptor 1 | Lpar1 | NM_010336 | 0.002862 | 1.22 |
| B | Ankyrin repeat and SOCS box-containing protein 8 | Asb8 | NM_030121 | 0.001412 | 1.22 |
| B | G protein-coupled receptor 26 | Gpr26 | NM_173410 | 0.000769 | 1.36 |
| B | Olfactory receptor 875 | Olfr875 | NM_146749 | 0.000365 | 1.46 |
| B | Required for meiotic nuclear division 1 homolog (S. cerevisiae) | Rmnd1 | NM_025343 | 0.000315 | 2.05 |
| C | Aldo-keto reductase family 1, member C13 | Akr1c13 | NM_013778 | 0.007878 | -1.46 |
| C | Sulfide quinone reductase-like (yeast) | Sqrdl | NM_021507 | 0.000167 | -1.21 |
| C | Embryonic ectoderm development | Eed | NM_021876 | 0.006592 | -1.24 |
| C | MOCO sulphurase C-terminal domain containing 2 | Mosc2 | NM_133684 | 0.008203 | -1.25 |
| C | UDP-GlcNAc:betaGal beta-1,3-N-acetylglucosaminyltransfera | B3gnt2 | NM_016888 | 0.003860 | -1.43 |
| C | Transmembrane protein 38B | Tmem38b | NM_028053 | 0.006098 | -1.24 |
| C | YKT6 homolog (S. Cerevisiae) | Ykt6 | NM_019661 | 0.005953 | -1.31 |
| C | Mediator of RNA polymerase II transcription, subunit 10 ho | Med10 | NM_138596 | 0.006268 | -1.44 |
| C | Coenzyme Q10 homolog B (S. cerevisiae) | Coq10b | NM_001039710 | 0.002869 | -1.28 |
| C | Sterile alpha motif domain containing 4B | Samd4b | NM_175021 | 0.004401 | -1.26 |
| C | Glutathione S-transferase, theta 2 | Gstt2 | NM_010361 | 0.006261 | -1.22 |
| C | Polo-like kinase 4 (Drosophila) | Plk4 | NM_011495 | 0.002464 | -1.33 |
| D | Coiled-coil domain containing 47 | Ccdc47 | NM_026009 | 0.004097 | -1.41 |
| D | Complement component 1, q subcomponent binding protein | C1qbp | NM_007573 | 0.006490 | -1.35 |
| D | Centrin 3 | Cetn3 | NM_007684 | 0.001501 | -1.46 |
| D | Proteasome (prosome, macropain) 26S subunit, non-ATPase, | Psmd14 | NM_021526 | 0.004170 | -1.27 |
| D | Glucan (1,4-alpha-), branching enzyme 1 | Gbe1 | NM_028803 | 0.005752 | -1.31 |
| D | Nuclear transcription factor-Y beta | Nfyb | NM_010914 | 0.000937 | -1.26 |
| D | Coiled-coil domain containing 43 | Ccdc43 | NM_025918 | 0.007952 | -1.21 |
| D | Olfactory receptor 512 | Olfr512 | NM_146724 | 0.002486 | -1.24 |
| D | RIKEN cDNA 2310056P07 gene | 2310056P07Rik | NM_027342 | 0.000739 | -1.29 |
| D | Solute carrier family 7, member 6 opposite strand | Slc7a6os | NM_001007567 | 0.003962 | -1.34 |
| D | Josephin domain containing 3 | Josd3 | NM_029248 | 0.008235 | -1.48 |
| D | Mitochondrial ribosomal protein S34 | Mrps34 | NM_023260 | 0.005382 | -1.25 |
| D | Coiled-coil-helix-coiled-coil-helix domain containing 4 | Chchd4 | NM_133928 | 0.006729 | -1.34 |
| D | Protein kinase C, beta 1 | Prkcb1 | NM_008855 | 0.004713 | -1.28 |
| D | Translocase of inner mitochondrial membrane 9 homolog (yeast) | Timm9 | NM_013896 | 0.005376 | -1.24 |
|  | Carbonyl reductase 3 | Cbr3 | NM_173047 | 0.005382 | -1.37 |
|  | Translocase of inner mitochondrial membrane 8 homolog b | Timm8b | NM_013897 | 0.009553 | -1.61 |
| ***Not shown*** | ***Arginase 1, liver*** | ***Arg1*** | ***NM_007482*** | ***0.016438*** | ***-3.11*** |
